# Integrated single-cell multiomics analysis reveals novel candidate markers for prognosis in human pancreatic ductal adenocarcinoma

**DOI:** 10.1038/s41421-021-00366-y

**Published:** 2022-02-15

**Authors:** Xiaoying Fan, Ping Lu, Hongwei Wang, Shuhui Bian, Xinglong Wu, Yu Zhang, Yang Liu, Danqi Fu, Lu Wen, Jihui Hao, Fuchou Tang

**Affiliations:** 1 Beijing Advanced Innovation Center for Genomics (ICG), School of Life Sciences, Peking University, Beijing, China; 2 The Fifth Affiliated Hospital of Guangzhou Medical University, Guangzhou, Guangdong China; 3 Department of Pancreatic Cancer, Tianjin Medical University Cancer Institute and Hospital, National Clinical Research Center for Cancer, Key Laboratory of Cancer Prevention and Therapy, Tianjin’s Clinical Research Center for Cancer, Tianjin, China; 4 Biomedical Pioneering Innovation Center, Ministry of Education Key Laboratory of Cell Proliferation and Differentiation, Beijing, China; 5 State Key Laboratory of Reproductive Medicine, Nanjing Medical University, Nanjing, Jiangsu China; 6 College of Animal Science and Technology, Hebei Agricultural University, Baoding, Hebei China; 7 Peking-Tsinghua Center for Life Sciences, Academy for Advanced Interdisciplinary Studies, Peking University, Beijing, China

**Keywords:** Cancer genomics, Methylation analysis, Gene expression profiling

## Abstract

The epigenomic abnormality of pancreatic ductal adenocarcinoma (PDAC) has rarely been investigated due to its strong heterogeneity. Here, we used single-cell multiomics sequencing to simultaneously analyze the DNA methylome, chromatin accessibility and transcriptome in individual tumor cells of PDAC patients. We identified normal epithelial cells in the tumor lesion, which have euploid genomes, normal patterns of DNA methylation, and chromatin accessibility. Using all these normal epithelial cells as controls, we determined that DNA demethylation in the cancer genome was strongly enriched in heterochromatin regions but depleted in euchromatin regions. There were stronger negative correlations between RNA expression and promoter DNA methylation in cancer cells compared to those in normal epithelial cells. Through in-depth integrated analyses, a set of novel candidate biomarkers were identified, including *ZNF667* and *ZNF667-AS1*, whose expressions were linked to a better prognosis for PDAC patients by affecting the proliferation of cancer cells. Our work systematically revealed the critical epigenomic features of cancer cells in PDAC patients at the single-cell level.

## Introduction

Pancreatic cancer, which is named the king of cancers, is highly lethal with extremely poor prognosis^1,2^. Pancreatic ductal adenocarcinoma (PDAC) is the most common type of pancreatic cancer, and surgical resection is the only chance for the cure; however, no more than 20% of PDAC patients are eligible for this treatment strategy^3^. Whole-genome analyses of PDAC tissues have uncovered key driver mutations, pathways, and subtypes^4–7^, and the DNA methylation analysis at bulk level revealed frequent hypomethylation of multiple genes, including the HOX cluster and histone core proteins^8,9^. Meanwhile, more genes are hypermethylated, such as *SMAD4*, *STAT4*, zinc finger proteins, and the SLIT-ROBO signaling pathway genes^9,10^, indicating extremely complicated regulation mechanisms. Single-cell sequencing studies have largely revealed intratumoral heterogeneity by gene expression profiles^11–13^. Especially, these studies demonstrated the characteristics of different types of cells in the tumor microenvironment, offering clues on the molecular changes of epithelial cells during cancer progression. A recent study explored the enhancer network in mouse pancreatic cancer model^14^, where they also included scATAC-seq data from a PDAC patient to show the difference in the chromatin status between normal and cancer cells. These single-cell epigenetic data from patients were quite limited for comprehensive analysis, especially for identifying the global features of PDAC cells. The epigenetic characteristics of PDAC cells remain largely elusive due to their extremely high intratumoral stromal content^15^.

Here, we improved our single-cell multiomics sequencing method^16^, integrating modified STRT-seq^17^ with scCOOL-seq^18^ to simultaneously assess the genome (copy number variations), DNA methylome, chromatin accessibility, and transcriptome in the same individual cell (see “Materials and methods” section). The integration of multiomics data set was performed through correlation analysis between every two modalities with exactly the same cell IDs^19^. We applied the technique to characterize epithelial cells inside the primary tumor tissues (Pris) and adjacent normal tissues (Adjs). We performed multiregional sampling for a total of 13 PDAC patients (stages I and II), generating 1295 high-quality single-cell multiomics profiles to fully investigate how multiple omics coordinate with each other to determine the heterogeneity of PDAC cells.

## Results

### Characterizing epigenome of normal epithelial cells in the primary tumor tissue

**Fig. 1:**
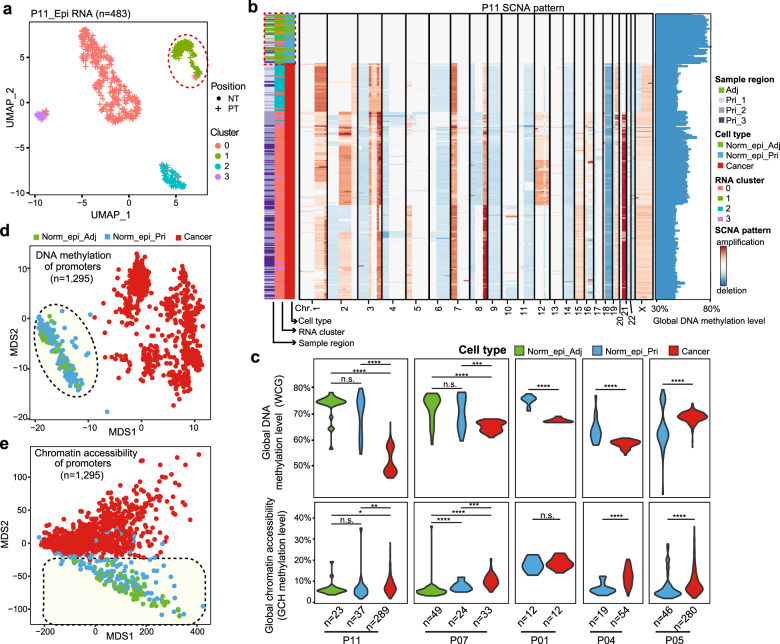
Normal epithelial cells were identified in the primary PDAC tissues. **a** Unsupervised UMAP showing the clustering of epithelial cells using the transcriptome of P11. The red dashed line indicates epithelial cells in primary tumor tissue in the same cluster as those in adjacent tissue. **b** SCNA map showing the high frequency of subchromosome-scale SCNAs in P11. The red dashed line indicates that a significant proportion of epithelial cells in the primary tissue regions have euploid genomes and show similarity to epithelial cells in the adjacent tissue in RNA expression patterns. Other patients are shown in Supplementary Fig. S4. The bar plot on the right shows the heterogeneity of the global DNA methylation level among the cells. **c** Violin plot showing the global DNA methylation level and chromatin accessibility in the Norm_epi_Pri cells, Norm_epi_Adj cells and cancer cells of each patient. The statistical test was carried out using the Wilcoxon rank-sum test. n.s., no significance; *P < 0.05; **P < 0.01; ***P < 0.001; ****P < 0.0001. Only patients with each type of epithelial cells >10 were shown. **d** MDS analysis of promoter DNA methylation levels for all epithelial cells (1295 cells) from all 13 patients. The shadow in the dashed line indicates the relatively uniform promoter DNA methylation pattern for the Norm_epi cells from both adjacent tissues and primary tumor tissues. **e** MDS analysis of promoter chromatin accessibility for all epithelial cells (1295 cells) from all 13 patients. The shadow in the dashed line indicates the differences between cancer cells and the two types of Norm_epi cells.

Single-cell multiomics sequencing technology was applied to epithelial cells in multiple regions of Pris and Adjs of 13 PDAC patients (Supplementary Figs. S1 and S2). To precisely analyze the cancer cells without being confounded by other types of cells, we first refined the epithelial cells by dimension reduction and cell clustering based on the gene expression profiles. Then we chose the corresponding nuclear fractions of these epithelial cells to perform scCOOL-seq one by one, generating high coverage data for each individual cell (Supplementary Fig. S1b, c and Table S1). Totally 3225 single cells from all patients were obtained for dimension reduction and cell clustering after quality control. The majority (89%) of the cells were confirmed to be epithelial cells, which were allocated into 11 clusters with most of the clusters showing patient-specific features, indicating strong inter-patient heterogeneity in the PDAC cells (Supplementary Fig. S3a). The remaining small part of the cells were non-epithelial cell types which were identified as macrophages, T cells and fibroblasts, showing consistent gene expression features among different patients as expected (Supplementary Fig. S3b, c). To reveal the genomic heterogeneity of cancer cells, we further performed subclustering of the epithelial cells in each patient (Fig. 1a and Supplementary Fig. S4). Interestingly, we observed that 29.6% (24 out of 81) and 8.4% (37 out of 438) of the epithelial cells from primary tumor tissues of P07 and P11, respectively, clustered together with those from adjacent normal tissues of the corresponding patients, indicating that primary tumor tissues may contain a significant proportion of non-cancer epithelial cells (Norm_epi) (Fig. 1b and Supplementary Fig. S4a, c). We further analyzed the somatic copy number alteration (SCNA) pattern in each individual cell and confirmed that these normal epithelial cells from Pri have euploid genomes, clearly different from the cancer cells with abundant SCNAs in the same patients (Fig. 1b and Supplementary Fig. S4).

From 8 out of these 13 patients, we obtained a significant proportion of Norm_epi cells with euploid genomes in the primary tumor lesions (Fig. 1b and Supplementary Figs. S4, S5, Table S1), and we named them Norm_epi_Pri cells. In addition, 88% (21 out of 24) and 41% (15 out of 37) of Norm_epi_Pri cells were clustered together with Norm_epi_Adj cells in P07 and P11, respectively, while the remaining cells were similar to cancer cells in gene expression. We further evaluated the DNA methylome and chromatin accessibility in these cells. We obtained scCOOL-seq data from Norm_epi_Adj in P07 and P11, and found comparable DNA methylation patterns and chromatin accessibility patterns between Norm_epi_Pri cells and Norm_epi_Adj cells (Fig. 1c). The cancer cells showed globally lower DNA methylation levels (5%–20% lower) than the Norm_epi cells in each patient except P05 in our data set (Fig. 1c). The chromatin accessibility was not uniformly increased for different patients (Fig. 1c). Using multidimensional scaling (MDS) of the DNA methylation levels at gene promoter regions, the Norm_epi_Pri cells clustered together with Norm_epi_Adj cells in all patients, indicating comparable promoter methylation patterns between these two types of cells. The cancer cells in different patients exhibited different DNA methylation patterns of promoter regions according to the rather dispersed distributions of these cells on the MDS map (Fig. 1d and Supplementary Fig. S6a). With regard to chromatin accessibility, although the global level in cancer cells varied for different patients (Fig. 1c), the Norm_epi_Adj and Norm_epi_Pri cells were clustered together but separated from the cancer cells based on the GCH methylation level in promoter regions, indicating comparable chromatin states between the Norm_epi_Adj and Norm_epi_Pri cells in gene promoter regions (Fig. 1e and Supplementary Fig. S6b).

To validate that the Norm_epi_Pri cells truly existed in tumor tissues but did not result from the potential experimental contaminations, we compared the gene expressions of the Norm_epi cells and the cancer cells within individual patients, and identified the specifically expressed genes in the Norm_epi cells (Supplementary Fig. S7a). Two genes were chosen to be general Norm_epi cell markers. One is *CTRB2*, which was reported to be associated with pancreas digestion function^20,21^ and the other gene *REG1A* could promote the acinar-to-ductal metaplasia (ADM) process^22–24^. We further did immunohistochemical staining of these two proteins in the tumor tissues of the patients we analyzed. Indeed, most of the patients’ tumor tissues contained *REG1A*- and *CTRB2*-positive cells, just around the cancer cells (Supplementary Fig. S7b). Previous studies also implied the existence of the relatively normal ductal cells (ductal 1 cells) in the tumor tissues of PDAC patients^11^. To reveal whether the Norm_epi_Pri cells identified by our single-cell multiomics analysis were similar to those normal ductal cells identified by the previous study, we compared these cell subsets according to their gene expression patterns (Supplementary Fig. S7c). Some of the Norm_epi_Pri cells clustered with Norm_epi_Adj cells whereas the remaining ones were more similar to cancer cells in gene expression, indicating that the Norm_epi_Pri cells are genetically euploid and epigenetically normal epithelial cells. Some of them showed normal RNA expression patterns, while the remaining ones showed RNA expression patterns more similar to their neighboring cancer cells, probably due to their cancer microenvironment.

### Aberrant DNA methylation in the gene body and promoter regions in the cancer cells

**Fig. 2:**
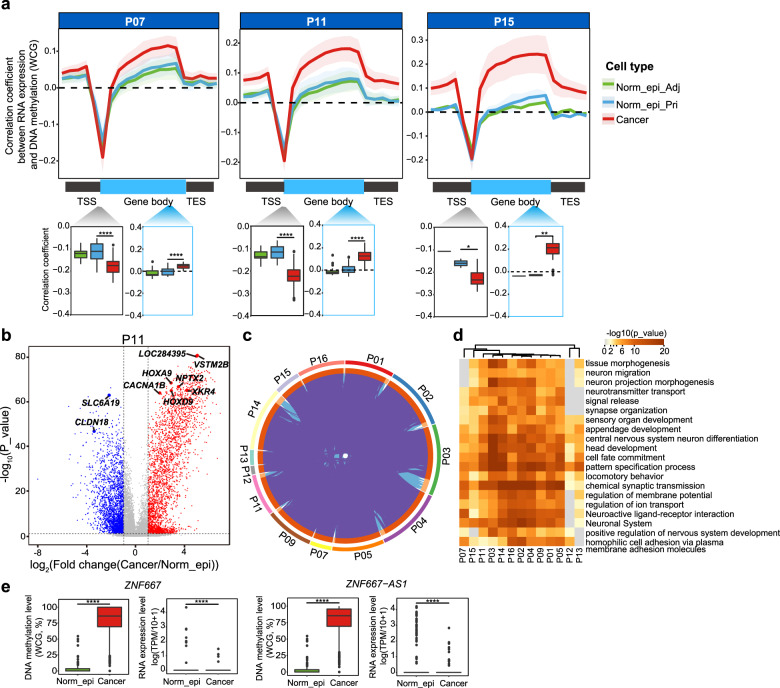
DNA methylation shows a stronger correlation with RNA expression in cancer cells. **a** Spearman correlations between DNA methylation levels across gene bodies (including 15 kb flanking regions) and corresponding RNA expression levels in each cell type in representative patients. The boxplots at the bottom show the statistical test results between cancer cells and Norm_epi cells using the Wilcoxon rank-sum test. The promoter regions are from –1 to +0.5 kb around the transcription start site (TSS), and the gene body regions are +2 kb from the TSS to the transcription end site (TES). *P < 0.05; **P < 0.01; ****P < 0.0001. **b** Cancer cells have more genes with increased promoter DNA methylation. Representative genes across multiple patients are labeled. **c** Overlap of higher methylated promoters across all patients. The purple lines indicate shared genes, and the blue lines represent the same Gene Ontology terms. **d** Heatmap showing the top enriched biological terms of the cancer cell hypermethylated promoters in each patient. **e** Example genes show a reversed pattern between the promoter DNA methylation level and RNA expression level. The statistical test was carried out using the Wilcoxon rank-sum test. ****P < 0.0001.

Since the Norm_epi_Pri cells showed consistent signatures with the Norm_epi_Adj cells for the genome, DNA methylome, chromatin accessibility, and for the majority of the cases, we could not obtain the matched Adjs, we set these cells as Norm_epi cell controls. When analyzing the cross-omics relations, we found that the positive correlation between RNA expression and corresponding gene body DNA methylation was clearly stronger in cancer cells than that in normal epithelial cells (Fig. 2a and Supplementary Fig. S8a). We further explored the DNA methylation changes in gene body regions for the genes with different expression levels. By dividing genes into four groups according to their expression levels in the Norm_epi cells, we found that genes with lower expression levels showed stronger DNA demethylation in gene body regions in the cancer cells (Supplementary Fig. S8b, c). These results indicate that the DNA hypomethylation of gene body regions may play potential roles in tumorigenesis in PDAC patients. We also found that the negative correlations between RNA expression and promoter DNA methylation of corresponding genes were much stronger in cancer cells than in Norm_epi cells, indicating that the hypermethylation of promoter regions may play role in tumorigenesis of PDAC (Fig. 2a and Supplementary Fig. S8a). We sought to identify the differentially methylated promoters between Norm_epi cells and cancer cells in each patient via stringent filtering criterion (see “Materials and methods” and Supplementary Table S2). All 13 patients showed more genes with increased promoter DNA methylation than genes with decreased promoter DNA methylation in cancer cells (Fig. 2b and Supplementary Fig. S9a), consistent with the global hypermethylation of promoter regions and CpG islands (CGIs) in PDAC patients^10^. Many of the hypermethylated gene promoters were shared across different patients, and they were enriched for genes related to the neural system by Gene Ontology (GO) analysis (Fig. 2c, d). This is consistent with the report that GO terms of genome aberrations and DNA methylation changes were largely enriched with the axon guidance pathway genes^7,10^, and dysregulation of these genes plays a role in tumor initiation and progression^25,26^. Although there were only 13–152 genes showing significantly lower promoter methylation levels in each patient, these genes were usually consistent across multiple patients (Supplementary Fig. S9b), indicating consistent demethylation mechanisms for these genes in cancer cells. A total of 53 genes were promoter hypomethylated in cancer cells of at least 3 PDAC patients, and these genes were enriched in GO terms such as transporting organic acids and metal ions, epithelial cell development, and negative regulation of endopeptidase activity (Supplementary Fig. S9c). The differentially methylated genes shared by all patients are labeled in Fig. 2b and Supplementary Table S2, and these genes could be potential DNA methylation biomarkers for PDAC. A subset of 77 differentially methylated promoters showed a clear negative correlation between promoter DNA methylation and gene expression in the cancer cells we analyzed (Supplementary Table S2). We further compared these candidate biomarkers to those reported by the previous studies based on abnormal DNA methylation levels^8–10,27,28^. A subset of genes was newly identified in our data set of integrated omics information (Supplementary Table S2). We confirmed that the DNA methylation of PDAC survival-associated genes such as the voltage-gated calcium channel gene *CACNA1B* was aberrantly regulated (Supplementary Fig. S9d). *XKR4* was also identified as a novel candidate marker for PDAC (Supplementary Fig. S9d). Furthermore, *ZNF667* and *ZNF667-AS1*, which were recently reported in laryngeal squamous cell carcinoma^29^, were identified as novel candidate markers for PDAC patients (Fig. 2e and Supplementary Fig. S9e).

### *ZNF667* and *ZNF667-AS1* as candidate novel markers for prognosis in PDAC

**Fig. 3:**
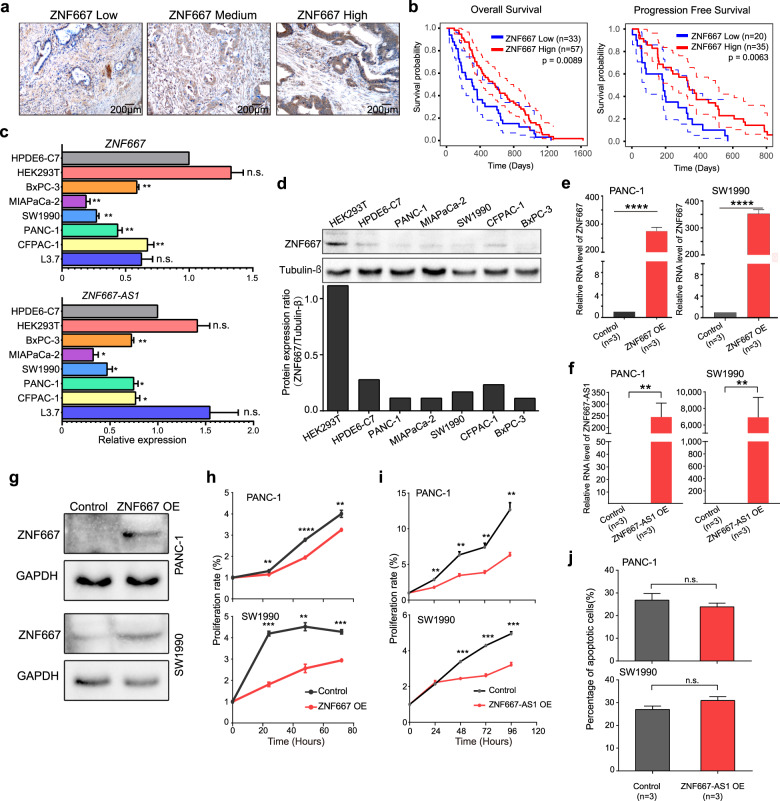
*ZNF667* and *ZNF667-AS1* as novel markers for prognosis in PDAC. **a** Immunohistochemistry of ZNF667 shows variations of abundance across patients using the tissue microarray analysis. **b** Overall survival and progression-free survival of self-collected PDAC patients grouped by the abundance of ZNF667. **c** Relative abundance of *ZNF667* and *ZNF667-AS1* to *ACTB* mRNA in normal pancreas cells (HPDE6-C7), human embryonic kidney cells (HEK293T), liver metastasis pancreas cancer cells (L3.7), and the pancreas cancer cells detected by RT-qPCR. All pancreas cancer cells showed a decreased level of *ZNF667* and *ZNF667-AS1*. *P < 0.05; **P < 0.01. **d** Immunoblot showing the decreased abundance of ZNF667 in multiple pancreas cancer cell lines. The abundance was calculated at the bottom. **e**, **f** Validation of *ZNF667* and *ZNF667-AS1* overexpression (OE) in two pancreas cancer cell lines. **P < 0.01; ****P < 0.0001. **g** Immunoblot showing the abundance of ZNF667 in PANC-1 and SW1990 cells before and after overexpression. **h**, **i** Proliferation evaluation (CCK8-positive cell ratio) after *ZNF667* and *ZNF667-AS1* overexpression in two pancreas cancer cell lines. *n* = 3 for each time point. **P < 0.001; ****P < 0.00001. **j** Evaluation of cellular apoptosis after *ZNF667-AS1* overexpression in two pancreas cancer cell lines. n.s., no significance.

*ZNF667* and *ZNF667-AS1* are head-to-head with each other on chromosome 19, shared a large fraction of the promoter region, but have no overlaps between these two transcripts (Supplementary Fig. S10a). To further investigate whether these two genes are potential markers for PDAC, we firstly did survival analysis using tissue microarrays with ZNF667 antibody on 98 PDAC patients and they showed heterogeneity of the protein abundance (Fig. 3a). As expected, both overall (from diagnosis to death) and progression-free (from surgery to relapse) survival time were significantly longer in patients with higher expression levels of ZNF667 (Fig. 3b), suggesting ZNF667 as an effective marker in prognosis diagnosis of PDAC. Moreover, the ZNF667 protein level was higher in normal pancreas tissue than in the PDAC tissue (Supplementary Fig. S10b), indicative of efficient biomarkers for PDAC detection.

Next, we interrogated whether *ZNF667* and *ZNF667-AS1* have functions in suppressing PDAC. We examined the expression levels of these two genes in multiple pancreatic cancer cell lines. Compared to the normal cell line of the human pancreas HPDE6-C7, all the pancreatic cancer cell lines we analyzed showed much lower RNA expression levels of *ZNF667* and *ZNF667-AS1*, except for L3.7, the cell line from a liver metastasis of pancreatic cancer (Fig. 3c). As a control, the decrease of RNA expression of these two genes was not observed in HEK293T, a human fetal kidney cell line (Fig. 3c). The ZNF667 protein was also less abundant in the pancreas cancer cell lines compared to that in control cell lines (Fig. 3d). Although the pancreatic cancer cell lines also showed significantly lower expression levels of these two genes, the methylation levels of their promoter regions in these cell lines in vitro were quite low, which was different from those we detected in the patients in vivo (Supplementary Fig. S10c, d). On the other hand, the promoter of these two genes in in vivo tumor tissues was verified as highly methylated by bisulfite PCR-coupled Sanger sequencing (Supplementary Fig. S10e), suggesting different regulation mechanisms of these two genes between in vitro cultured cell lines and in vivo cancer cells in patients.

To investigate the potential functions of *ZNF667* and *ZNF667-AS1* in suppressing pancreas tumorigenesis, we separately overexpressed these two genes in two cancer cell lines, PANC-1 and SW1990 (Fig. 3e–g). These two genes showed inter-dependent expressions (Supplementary Fig. S11a). By analyzing the CCK8-positive cell ratios, we found that when overexpressing either *ZNF667* or *ZNF667-AS1*, the proliferation of both cell lines were slowed down (Fig. 3h, i). This suggested that both of these two genes suppress the proliferation of pancreas cancer cells. In addition, we calculated the correlation between the expression levels of these two genes and the cell cycle genes in pancreas cancer bulk samples in TCGA. Both genes were negatively correlated with the cell cycle genes in expression levels (Supplementary Fig. S11b). On the other hand, we also evaluated apoptosis after overexpressing *ZNF667* and *ZNF667-AS1* in both cancer cell lines (Supplementary Fig. S11c, d). In both cell lines, neither genes affected apoptosis in general (Fig. 3j). Together, these results indicated that *ZNF667* and *ZNF667-AS1* both suppress tumorigenesis of PDAC via suppressing proliferation but not via promoting apoptosis of cancer cells.

### Subclones exist within PDAC primary tissue and global DNA demethylation is enriched in heterochromatin regions

The different SCNA patterns in the cancer cells could indicate the emergence of subclones that occur during tumorigenesis^16,30^. We then investigated the subclones in patients with over 200 individual cancer cells sequenced using SCNA clustering. The globally similar SCNAs in all cells indicated a common genetic origin for cancer cells in the same patient. A total of 289 cancer cells in P11 could be clearly clustered into 3 subclones (Supplementary Fig. S12a, b). We observed several subclonal SCNAs that also appeared in some cancer cells belonging to other subclones, indicating that reversible or convergent changes of SCNAs may occur in the genomic regions during tumorigenesis, making it difficult to trace the cancer cell lineage derivation (Supplementary Fig. 12a). This phenomenon was also observed in the cancer cells of P05 (Supplementary Fig. S12d, e), indicating the extremely unstable genome in PDAC cells. As expected, the same tumor regions contain cancer cells from different subclones, but just with variable proportions, indicating potential intratumoral migration and mixing between different cancer subclones (Supplementary Fig. S12c).

**Fig. 4:**
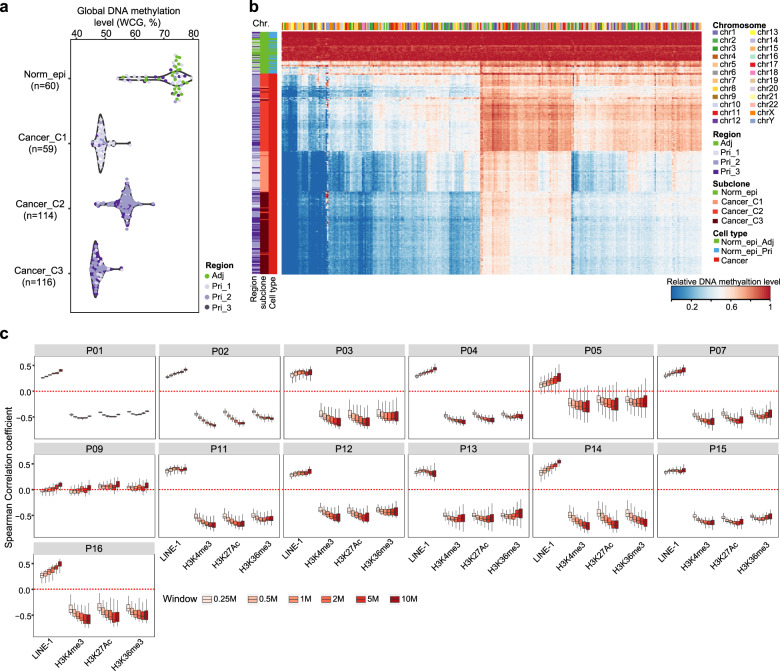
Genome-wide DNA demethylation in cancer cells is strongly enriched in heterochromatin regions. **a** Global DNA methylation levels (1 kb tile) of single cells in each subclone of P11. **b** Genome-wide DNA demethylation heatmap (10 Mb window) of cancer cells compared to all Norm_epi cells in P11. **c** Spearman correlations between DNA demethylation levels and the densities of genomic landmarks under different resolutions in each patient.

Since the cancer cells from P11 exhibited highly heterogeneous DNA hypomethylation (Fig. 1b), we further investigated whether the heterogeneity of DNA methylation was mainly between different subclones or within the same subclone. Indeed, the cells in subclone1 and subclone3 showed 10% lower DNA methylation levels than those in subclone2 (Fig. 4a). Therefore, in PDAC, the DNA methylation levels of cancer cells within the same subclone are usually similar but can be quite different between different subclones (Fig. 4b). The changes of DNA methylation levels were not correlated with the SCNAs in each subclone (Supplementary Fig. S13), indicating that SCNA changes do not seem to affect the DNA methylation of corresponding genomic regions.

In general, the PDAC cells in the same patient experienced variable degrees of genome-wide DNA demethylation compared to the Norm_epi cells (except for P05 in the study) (Figs. 1c and 4b). Since the repeat element long interspersed element-1 (LINE-1) contributes ~17% of the genome^31^, it is reasonable that it showed the decreased methylation levels consistent with the whole-genome patterns. On the contrary, the genomic regions such as CGIs, promoters, and exon regions tend to have increased levels of DNA methylation (Supplementary Fig. S14). Importantly, the DNA demethylation levels in cancer cells were positively correlated with the densities of LINE-1 but negatively correlated with densities of H3K4me3, H3K27Ac and H3K36me3 (Fig. 4c), indicating that the DNA demethylation in PDAC cells was strongly enriched in the heterochromatin regions (LINE-1-enriched regions), but depleted in the euchromatin regions (H3K4me3-, H3K27Ac-, or H3K36me3-marked regions)^32^. These results suggested that PDAC tumorigenesis might involve the heterochromatin disorganization, including their prevalent DNA demethylation, similar to the situation in colorectal cancer, as we previously showed^16^.

### Open chromatin regions and corresponding transcription factors (TFs) in PDAC tumorigenesis

**Fig. 5:**
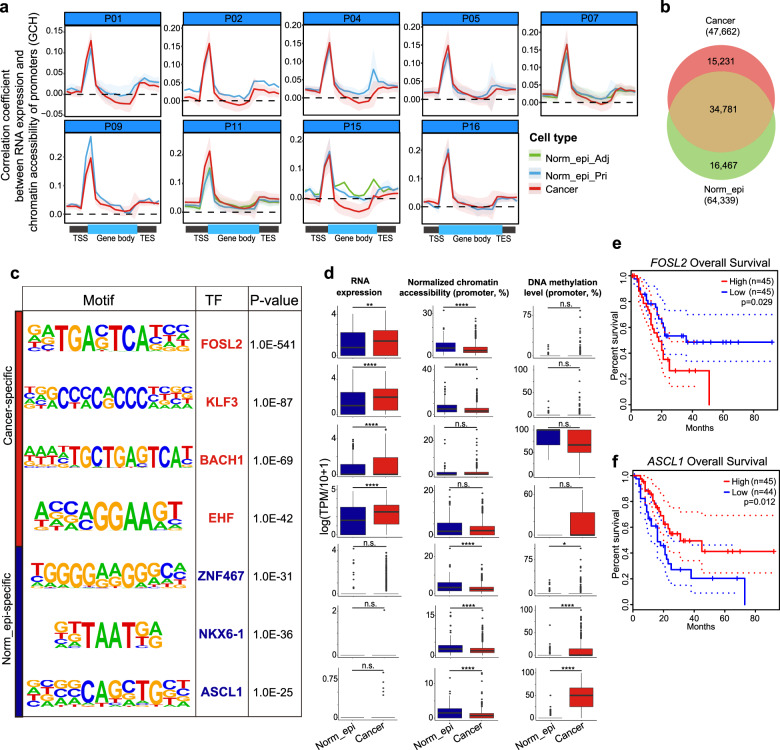
NDRs and TF motif enrichment in PDAC. **a** Spearman correlations between chromatin accessibility across gene bodies (including 15 kb flanking regions) and corresponding RNA expression levels in each cell type. **b** Venn diagram indicating the NDR numbers in cancer cells and Norm_epi cells. NDRs in two types of cells are defined as a shared NDR when they have at least a 100-bp overlap. Merged NDRs in two types of cells were defined with two standards: (1) NDRs were merged when they had at least a 100-bp overlap; (2) each merged NDR was detected in at least 11 cells in Norm_epi cell group and 53 cells in the cancer cell group. **c** Representative cancer-specific and Norm_epi-specific motifs and corresponding candidate TFs. **d** RNA expression levels, chromatin accessibility, and DNA methylation levels in the promoter regions of each candidate TF. The statistical test was carried out using the Wilcoxon rank-sum test. n.s., no significance; *P < 0.05; **P < 0.01; ***P < 0.001; ****P < 0.0001. **e**, **f** Overall survival of PDAC patients grouped by two representative genes. The survival data were obtained from the TCGA database.

As we obtained the pairwise chromatin accessibility (GCH methylation level), we examined how this epigenetic regulation affected gene expression levels. As expected, the extent of open chromatin states in promoter regions clearly positively correlated with the RNA expression levels of the corresponding genes in all cell types we analyzed (Fig. 5a). Despite the disparity in the cell numbers of the Norm_epi cell group and the cancer cell group, we used the same cutoff (detected in at least 11 individual cells in each group) and identified 64,339 and 265,213 nucleosome-depleted regions (NDRs, the genomic regions with local open chromatin features) in Norm_epi cells and cancer cells, respectively (Supplementary Fig. S15a; see “Materials and methods”). Then we also identified the shared open chromatin regions between different patients by analyzing NDRs shared by at least one-third of the cells in each group, and identified 64,339 and 47,622 NDRs in Norm_epi cells and cancer cells, respectively, of which ~70% were shared by both groups (Fig. 5b). This indicated that the majority of the cancer-specific NDRs were individual patient-specific and only a small percentage of the cancer-specific NDRs were shared between different patients. A previous study has performed scATAC-seq in limited PDAC patient samples^14^. To evaluate the accuracy of our data, we compared the open chromatin sites identified in normal and cancer cells between both studies. With a much-enlarged sample size and a more sensitive detection method, we captured many more open chromatin sites in each cell type (Supplementary Fig. S15b). Furthermore, 95.6% of the open chromatin sites identified in the scATAC-seq of cancer cells were the same as those in our scCOOL-seq of cancer cells. 88.8% of the open chromatin sites identified in the scATAC-seq of normal epithelial cells were the same as those in our scCOOL-seq of normal epithelial cells (Supplementary Fig. S15b). These results proved the reliability of our data with higher sensitivity. We further did GO analysis of the top 200 cell type-specific NDR-related genes (Supplementary Fig. S15c, d). Interestingly, the neural-related terms, which showed significant enrichment in the cancer hypermethylated genes, were enriched in the open chromatin sites of Norm_epi cells, revealing that their mis-regulation on different omic layers was potentially involved in tumorigenesis of PDAC.

*Cis*-regulatory elements are important in maintaining cell identities due to their typical binding with TFs^33^. We further searched for significantly enriched TF binding motifs using cancer-specific and Norm_epi-specific NDRs separately and identified candidate regulatory TFs potentially promoting or suppressing PDAC tumors (Fig. 5c and Supplementary Table S3). Enrichment of TFs proved important to drive PDAC progression, such as AP-1 factors and E2F TFs^14^, was also observed in our data set (Supplementary Table S3). The motifs of the Kruppel-like factor subfamily genes such as *KLF1/3/4/5/6/14* were specifically enriched in the NDRs in the cancer cells^34^. Some of the TFs with specifically enriched motifs in the NDRs in cancer or Norm_epi cells also showed significantly higher levels of RNA expression or promoter chromatin accessibility, or lower levels of promoter methylation in the corresponding cells (Fig. 5c, d and Supplementary Table S3). Moreover, their corresponding RNA expression showed clear positive correlations in both Norm_epi and cancer cells (Fig. 5d). Expressions of a couple of cell type-specific TFs were further validated in additional 6 sample pairs of PDAC patients by RT-qPCR (Supplementary Fig. S15e). These genes, such as *KLF3*^35^, *FOSL1,* and *FOSL2*^36^ (Fig. 5c and Supplementary Table S3), might play regulatory roles in PDAC progression. *FOSL2*, which showed higher RNA expression in cancer cells, was also significantly correlated with the poorer overall survival of the patients (Fig. 5d, e). Only less than half of these TFs showed expression profiles matching the DNA methylation changes at their promoter regions (Fig. 5d and Supplementary Table S3), implying the presence of regulation layers other than DNA methylation to regulate expressions of these TFs in PDAC patients.

Notably, the neural development-related TFs, which showed consistent promoter hypermethylation in cancer cells of all patients (Fig. 2d and Supplementary Table S2), also enriched binding motifs in the normal cells, such as *ASCL1*, *CUX1/2*, *DLX1/2/3/5*, *NEUROD1*, *NKX6-1*, *NEUROG2* (Fig. 5c and Supplementary Fig. S15c, Table S3), suggesting that dysregulation of these TFs may be involved in tumorigenesis of PDAC. More importantly, due to low expressions of these genes, we did not detect significant RNA expression changes according to the single-cell transcriptome data (Fig. 5d), indicating the importance of deducing gene functions in tumorigenesis according to their epigenetic features. Consistent with the inference, we observed longer overall survival of patients with higher expression of *ASCL1* although we did not detect differences in RNA levels between normal and cancer cells (Fig. 5f). Thus, we offered multiple layers of information to systematically search candidate genes associated with PDAC tumorigenesis.

## Discussion

There are strong heterogeneities of cancer cells in PDAC patients regarding the genome, epigenome, and transcriptome. More importantly, the heterogeneity within one omics layer may have complex relationships with the heterogeneity within another omics layer. For example, in the tumor of a PDAC patient, one genetic subclone may contain several different transcriptome subclusters with different gene expression features. Thus, the joint profiling of multiple omics in the same individual cell allows us to precisely elucidate the characteristics and relationships between these omics layers in tumors. We could interpret the epigenomic heterogeneities in different genetic subclones inferred by the SCNA patterns. In general, the SCNAs were not correlated with the DNA methylation changes, indicating that SCNA changes do not seem to affect DNA methylation of corresponding genomic regions in PDAC patients. At the same time, the transcriptome of the cells was used as the functional readout of the epigenome, including both DNA methylome and chromatin accessibility.

Different from previous studies that only determine normal cells by RNA expressions^11,14^, we used multiomics data to precisely define the non-cancer epithelial cells. In this way, we obtained significant proportions of cells as “normal epithelial cells” inside the PDAC tumor lesions, which exhibited normal diploid genome and DNA methylation and chromatin accessibility features comparable to those of normal epithelial cells in the tumor-adjacent tissues. However, the transcriptome statuses of these cells were quite diverse, ranging from similar to normal cells to similar to cancer cells. Thus, some of these cells were somehow different from those normal ductal cells identified in previous studies^11,14^, which represent normal epithelial cells with their transcriptome probably affected by their neighboring cancer cells, resulting in ADM characters. According to the immunohistochemical staining of normal cell-specific genes *CTRB1* and *REG1A*, these cells showed rather gathered patterns adjacent to cancer cells within the tumor lesions, and their distributions varied between different patients. Some of these normal cells also showed ADM characters. Due to the limited sample size for all the Norm_epi cells, it is inadequate to determine and analyze their subclusters. Future studies can focus on this issue and this may help to illustrate how the normal cells transformed into cancer cells in multiomics scale.

Although the gene expressions and SCNA patterns showed dynamic changes for different patients, the DNA methylation levels in CGIs and gene promoter regions were generally elevated in cancer cells in all the PDAC patients. The general features of PDAC cancer cells across multiple patients would be more valuable for clinical applications. All the patients showed elevated DNA methylation in promoter regions of the genes related to the neuronal system. Consistent with this, the promoter regions of such genes were in a closed chromatin state in the cancer cells. We believe that these interesting findings are meaningful for a deeper understanding of the molecular regulations in PDAC progression. However, this data set is limited in revealing additional mechanisms regarding PDAC tumorigenesis, and further investigations are needed to uncover the detailed mechanisms initiating these molecular progresses. It is interesting that the tumor subclones showed distinct DNA methylation patterns and their distribution within the tumor exhibited regional enrichment. However, we collected a relatively large number of cancer cells from each tumor region of only two patients in this cohort for analysis. Thus, the conclusions shall be confirmed with larger data set in the future.

The DNA methylation exhibited consistent global decreases in the majority of the PDAC patients, which was also implied by a series of studies in recent years^8,9,27,28,37^. Except for the focus on differentially methylated region analysis, we directly showed the global changes of DNA methylation precisely in each individual cell within each PDAC patient. We examined the DNA methylation and the correlated density of the hallmarks of chromatin state in the same individual cells, which gave us a more accurate and more comprehensive landscape of DNA methylation changes in PDAC cells.

With an integrated comparison of promoter methylation, chromatin accessibility, and RNA expression, we identified a couple of known and novel prognosis markers for PDAC, in which *ZNF667* and *ZNF667-AS1* were proved to play important roles in suppressing the proliferation of PDAC cells. Nevertheless, these two genes could only be regarded as candidate biomarkers as the verification of biomarkers needs more comprehensive data collection in a larger cohort, and tens of patients in our study could only be a clue to support further studies. On the other hand, the DNA methylation levels in gene body regions were decreased in the cancer cells, and the demethylation was stronger for lower expressed genes, enhancing the positive correlations between gene expression and gene body methylation in PDAC cells. Moreover, during tumor progression in both PDAC and colorectal cancer types, aberrant DNA demethylation favorably happened in heterochromatin regions, reflecting the heterochromatin disorganization also as an important feature of PDAC tumorigenesis.

In summary, our work offers novel insights into the molecular characteristics of PDAC tumorigenesis, and identifies candidate marker genes for the prognosis of PDAC through integrated single-cell multiomics analyses of PDAC patients.

## Materials and methods

### Processing of human tumor samples

This research was approved by the Ethics Committee of Tianjin Medical University Cancer Institute & Hospital (License# Ek2017141). Informed consent was obtained from all of the patients before surgery and chemotherapy. All of the patients in the study had not been treated before the surgery. Two to three separated regions from each resected pancreatic tumor were collected. Tumors and adjacent tissue were immediately processed in half an hour after resection. After being stripped of fat tissue, the tumors and adjacent tissue were cut into small pieces with sterile scissors and then digested using 1 mg/mL each of collagenase type II (Gibco, Cat# 17101015) and collagenase type IV (Gibco, Cat# 17104019). The digested content was passed through 40 μm cell strainers (CORNING Falcon, Cat# 352340). The epithelial cells from pancreatic cancer tissues and tumor-adjacent tissues were isolated by magnetic-activated cell sorting (MACS) (CD326 EpCAM MicroBeads, human, Cat# 130-061-101).

### Single-cell in vitro methylation

After collecting suspensions of EpCAM-positive cells isolated by MACS, we prepared a 2.5 µL cell lysis and in vitro methylation (IVM) mixture containing 4 U RNase inhibitor (Takara, Cat# 2313B), 0.25% IGEPAL CA-630 (Sigma, Cat# I3021), 1× GC reaction buffer and 2 U M.CviPI (NEB, Cat# M0227L). An individual cell was placed into the 2.5 µL IVM mixture and then gently vortexed for 10 s before running the IVM reaction on the thermocycler at 37 °C for 20 min and then at 65 °C for 25 min. Then, the cell lysate could be directly processed for RNA/DNA separation or stored at −80 °C.

### Single-cell RNA–DNA separation and cDNA library construction

We prepared a 3 µL nuclear separation mixture for each cell, containing 0.2 µL of Dynabeads Myone Carboxylic Acid (Invitrogen, Cat# 65011), 4 U RNase inhibitor, 0.2% Tween-20 (Sigma, Cat# P1379), 1% Triton X-100 (Sigma, Cat# T8787), 50 mM DTT and 2 µL of 5× Superscript II first-strand buffer (Invitrogen, Cat# 18064071). We added the mixture to the 2.5 µL cell lysate, mixed by vortexing, briefly centrifuged the tubes, and placed the tubes on an ice-cold 0.2 mL magnetic rack. The cell nuclei would be wrapped in the magnetic beads and attracted on the side of the tube, while the RNA is in the supernatant. We transferred the supernatant into a 4.5 µL reverse transcription (RT) mixture containing 4 U RNase inhibitor, 100 U SuperScript II reverse transcriptase (Invitrogen, Cat# 18064071), 1 mM dNTPs (Takara, Cat# 4019), 60 mM MgCl_2_, 3 µM RT primer with a 6-bp barcode and 10 µM TSO primer^17^. The RT reaction was directly carried out by incubation at 25 °C for 5 min, 42 °C for 60 min, 50 °C for 30 min, and 70 °C for 10 min in a thermocycler. Then, we performed cDNA amplification as previously reported^38,39^, but in a doubled volume of reaction. The cDNAs of different barcode sequences were pooled for downstream library construction and sequencing with the same method as described in previous studies^38,39^.

### Single-cell DNA library construction and sequencing

Only epithelial cells confirmed by transcriptome analysis were further selected for DNA library construction. We used 5 µL of protein lysis buffer containing 2.5 µL of M-digestion buffer (Zymo, Cat# D5044) and 0.5 µL of protease K (NEB, Cat# P8107S) to resuspend the bead-trapped nuclei. The genomic DNA in each cell was released after incubation at 50 °C for 1 h. Then, the genomic DNA lysates were stored at −80 °C, and we selected the genomic DNA of cells that were classified as epithelial cells through transcriptome analysis to perform DNA amplification. In brief, bisulfite conversion was carried out using the EZ-96 DNA Methylation-Direct™ Mag Prep Kit (Zymo, Cat# D5044). Specifically, we added only 32.5 µL of CT conversion reagent to 5 µL of single-cell genomic DNA lysate. We followed the steps of the single-cell whole-genome bisulfite sequencing workflow^40^ with minor modifications, including (1) We used random primers containing N6 sequences. (2) We performed a total of 4 rounds of olig1 tagging and skipped the Exo I digestion; instead, we removed the free primers by purification with 0.8 volume of Ampure XP beads (Beckman, Cat# A63882). Finally, we performed 16 cycles of the indexing PCR program at 98 °C for 15 s, 65 °C for 30 s, and 72 °C for 1 min. The DNA library for each cell was sequenced for 2 Gb (~0.6×) on the Illumina HiSeq 4000 platform.

### Processing of the single-cell RNA-seq data from multiomics sequencing

We used UMI-tools (version 0.5.5)^41^ to extract the single-cell information from the raw sequencing data of each library, including the cell barcodes (8 bp, 96 barcodes in total) followed by UMIs (8 bp random nucleotides) at the beginning of Read 2. Afterward, we used fastp (version 0.19.8)^42^ with default parameters and custom scripts to trim low-quality bases, adaptors, poly (A), and TSO sequences (AAGCAGTGGTATCAACGCAGAGTAC) in Read 1. The reads after trimming were further mapped to the human genome (hg19 in UCSC) using STAR (version 2.6.1d)^43^. Then, we used featureCounts from subread (version 1.6.3)^44^ to align the reads to RefSeq genes. UMI-tools (version 0.5.5) was used to count the UMI numbers of each gene with the parameter “-per-gene -gene-tag=XT -per-cell -wide-format-cell-counts”.

We filtered out single cells with <1500 detected genes or >9000 detected genes or 400,000 UMIs. A total of 3225 cells were retained for further analysis using Seurat (v3.0.0)^45^ to identify cell clusters, with the parameter “dims=1:15”. The cell types of each cluster were identified according to cluster-specific gene expression and the following known markers: *AIF1*, *CSF1R*, *CD86*, and *CD68* for macrophages; *PTPRC* (also known as *CD45*), *CD3D*, *CD69,* and *CXCR4* for T cells; *THY1*, *DCN*, *COL1A1*, *FN1*, and *LUM* for fibroblasts; and *EPCAM*, *KRT19*, and *MUC1* for epithelial cells^11^. A total of 11 clusters highly expressed the epithelial marker genes. We defined cells in the 11 clusters as epithelial cells. These cells were used for further analyses.

### Processing of single-cell DNA methylation data from multiomics sequencing

We used Trim Galore (version 0.4.4) (http://www.bioinformatics.babraham.ac.uk/projects/trim_galore/) to trim low-quality bases and random primers (6 bp) with the parameter “-quality 20 -stringency 3 -length 50 -clip_R1 6 -clip_R2 6 -paired -trim1 -phred33 -gzip”. Then, the clean data were mapped to the human genome (hg19, UCSC) as well as the lambda DNA genome reference using Bismark (version 0.7.6)^46^. The duplicate reads were removed using SAMtools (version 0.1.18)^47^. The cells that did not pass the strict quality control criteria (whole-genome coverage ratio ≥ 4%; number of WCG sites ≥ 800,000; number of GCH sites ≥ 5,000,000; mapping ratio ≥ 5%; and CT conversion rate ≥ 98%) were filtered out.

The methylation levels of WCG sites represent the endogenous DNA methylation levels. The in vitro DNA methylation levels of GCH sites represent chromatin accessibility. Only WCG sites with methylation levels >0.9 or <0.1 and GCH sites with methylation levels >0.9 or <0.1 were used. Promoters are defined as the upstream 1-kb regions and downstream 0.5-kb regions of the TSSs. We downloaded the annotation of repeat elements from the UCSC genome browser (http://genome.ucsc.edu/). When we calculated the DNA methylation levels or chromatin accessibility of genomic elements and 1-kb consecutive tiles, only the windows covered by ≥3 WCG or ≥3 GCH sites were used. To evaluate the whole-genome DNA methylation levels and chromatin accessibility of individual cells, we calculated the mean methylation levels for WCG or GCH sites in each 1-kb tile and used the averaged levels of the tiles to represent the global levels. The chromatin accessibility of the genomic elements (including promoters, CGIs, repeat elements, etc.) of each individual cell was further normalized by its global chromatin accessibility.

We performed classical MDS analysis using the DNA methylation levels and chromatin accessibility of gene promoters respectively with the R function “cmdscale” in R. Only the promoters covered by ≥3 WCG sites (for DNA methylation data) or ≥3 GCH sites (for chromatin accessibility data) were used in each individual cell. The MDS analysis finally used promoters covered in at least 50% of the cells that were used in this analysis.

To find the differentially methylated promoters between normal epithelial cells and cancer cells, the promoters covered (with ≥3 WCG sites) in <30% cells were filtered out. Then, we used strict criteria to reduce the number of false positives: *P*-value < 0.05 (Wilcoxon rank-sum test), fold change >2 or <0.5, absolute methylation differences between two groups >0.5, and stand deviation within each group <0.25.

### SCNA estimation using DNA sequencing data from single-cell multiomics sequencing

We deduced the copy number profiles in each individual cell using the Ginkgo tool^48^, as previously reported for colorectal cancer^16^. Briefly, the genome was binned into 2706 variable-length intervals with a median length of 1 Mb. A total of 72 normal epithelial cells from the male patients were used as controls for normalization. Under normal conditions, a male cell contains one copy of the X chromosome and one copy of the Y chromosome. Thus, we divided the values of the X and Y chromosomes of cells from both male and female patients by 2. Specifically, for visualization purposes, in the heatmaps of single-cell SCNA profiles, the colors represented states compared with normal conditions. Red represents amplifications, whereas blue represents deletions compared with normal conditions. For example, for cells from male patients, having one copy of the X chromosome is normal and therefore shown in white. The sex information for each patient is shown in Supplementary Table S1. Epithelial cells from the tumor lesions with obvious SCNA were defined as cancer cells, while the normal_epithelial cells showed a normal diploid pattern.

### Correlation calculation between RNA expression, DNA methylation, and chromatin accessibility

Each gene was divided into 20 equal-size windows from the TSS to the TES. The upstream 15-kb regions of the TSS were divided into 5 windows, and the downstream 15-kb regions of the TES were divided into 5 windows. For every window of each gene in every individual cell, we calculated the pairwise Spearman correlation between the RNA expression value and WCG methylation level, the RNA expression value and GCH methylation level, and the WCG methylation level and GCH methylation level. In addition, we also calculated these three types of Spearman correlations in whole promoter regions and the major gene body regions (from downstream 2-kb regions of TSSs to TESs). During this process, we filtered out the genes <2.5 kb.

### Immunohistochemistry and survival analysis for tissue microarray

The tumor tissues of 98 PDAC patients were affirmed by a pathologist from Tianjin Medical University Cancer Institute & Hospital and used to manufacture tissue microarray. Immunohistochemistry was performed according to the standard protocol. Briefly, antigen was retrieved by citrate sodium antigen retrieval buffer in high pressure and high-temperature condition after dewaxing. Tissue Microarrays (TMAs) were incubated with ZNF667 antibody (Novus, NBP1-77357), and then the sections were incubated with an immunohistochemistry kit from ZSGB-bio, and staining was detected. *H*-score was used for assessing protein expression by intensity and area of staining. The patients with *H*-scores of 6–9 were defined as ZNF667-high group, and those with *H*-scores of 0–4 were ZNF667-low group. After this step, the clinical survival data of different groups of patients were extracted and SPSS 26.0 was used to analyze the survival curve of the patients.

### Plasmid construction

We extracted the total RNA from the pancreas tissue using the QIAGEN RNeasy kit according to the manufacturer’s instructions. *ZNF667-AS1* was amplified by RT-PCR (forward primer: CCCTCGAGCGGTGTTGCGCCTGCGTAGCCG; reverse primer: GGAATTCGTCATGAGAAGGTGATTTATTGGAAAGT), and then *Xho*I (NEB, R0416) and *Eco*RI (NEB, R0101) were used to clone the cDNA fragments into the pEGFP-C1 backbone (Clonetech). *ZNF667* was amplified by PCR (forward primer: GCTCTAGAGCCACCATGCCTTCTGCACGGGGGAA, reverse primer: CGACGCGTTTAGGCTTTTTCTTCAGAATGT), and then *Xba*I (NEB, R0415) and *Mlu*I (NEB, R0198) were used to clone cDNA fragments into the pLV-MCS-bsd backbone (Biosettia). The reconstructed plasmids were extracted following the kit manufacturer’s instructions and were validated by Sanger sequencing.

### Culture and transfection of pancreatic cancer cell lines

PANC-1 and SW1990 cell lines are both ordered from ATCC. PANC-1 cells were cultured in DMEM supplemented with FBS to a final concentration of 10%. SW1990 cells were cultured in RPMI 1640 medium supplemented with FBS to a final concentration of 10%. For transfections, the PANC-1 or SW1990 cells were seeded at a density of 8 × 10^5^ cells per well in 6-well plates. After adherence, 2 μg plasmid of pEGFP-C1-CTRL or pEGFP-C1-ZNF667-AS1 together with 5 μL of lipofectamine 2000 (Thermo Fisher, 11668027) were added into each well, and the supernatants were replaced by DMEM or RMPI 1640 containing 10% FBS after 4 h. The transfected cells at different time points were digested using 0.05% trypsin for the following experiments.

### RT-qPCR

Total RNAs of the cell lines and the pancreas tissues were extracted using TRIZOL following the manufacturer’s instructions and cDNAs were obtained using PrimeScript RT Master Mix. RT-qPCR was performed using TB Green Premix Ex Taq in triplicate in three independent experiments on the Bio-Rad CFX Connect PCR system. *ACTB* was used as an endogenous normalization control. The qPCR primers are: *ZNF667*: forward-TTGGAGAATTACCGGAACCT, reverse-TCTTCTTACTGGCTCTACCAT; *ZNF667-AS1*: forward-CATCACTACCATCCATCACTA, reverse-CCAGGCAGAGAAGGATAA; *ACTB*: forward-TGGCACCACACCTTCTACAA, reverse-CCAGAGGCGTACAGGGATAG.

### Immunoblotting

Cells were lysed using 10% SDS lysis buffer with Protease Inhibitor Cocktail (Merck, P8340) and protein concentration was determined using Pierce BCA Protein Assay Kit (Thermo, 23225). 20 μg proteins were subjected to SDS-PAGE and transferred to the PVDF membrane according to standard protocols. After being blocked by nonfat milk, membranes were immunoblotted with antibodies against ZNF667 (Novus, NBP1-77357) and GAPDH (Proteintech, 10494-1-AP) overnight, and then incubated with ECL IgG Rabbit second antibodies (Proteintech, SA00002-2) before Bio-Rad Gel Doc XR + system was used for imaging.

### Detection of apoptosis and cell viability in pancreas cancer cells

PANC-1 and SW1990 cells were seeded at a density of 4000 cells per well in a 96-well plate after being transfected with pEGFP-C1-ZNF667 or pEGFP-C1-ZNF667-AS1. The medium was refreshed every other day, and then the relative viable cell counts were determined by the CCK8 assay (Bimake, B34304) every 24 h.

The apoptosis rate of cells was detected using FITC Annexin V Apoptosis Detection Kit (BD 556547) from BD Biosciences following the manufacturer’s instructions. Briefly, cells transfected with pEGFP-C1-CTRL, pEGFP-C1-ZNF667, or pEGFP-C1-ZNF667-AS1 were washed twice with cold PBS. 1 × 10^5^ cells were transferred to another culture tube and incubated with 5 μL of FITC Annexin V and PI in dark. Fluorescence cell sorting was performed using Mindray BriCyte E6 system and the results were analyzed using Flowjo V10.

### Correlations between DNA demethylation and histone modifications and LINE-1 density

We downloaded the ChIP-seq peak files of a 34-year-old male adult from the ENCODE website (https://www.encodeproject.org/), including data of H3K4me3 (ENCFF340YEE), H3K27Ac (ENCFF583QFI), and H3K36me3 (ENCFF544VYY). The genomic coordinates were cut into windows of equal length at different resolutions (0.25–10 Mb). For each type of window, the densities of genomic features (including histone modifications and LINE-1) were calculated as the length of overlap between the window and genomic features divided by window length; the DNA methylation level was calculated based on the DNA methylation level in normal and cancer cells. Because a subgroup of normal cells in P05 showed extremely low DNA methylation levels, we calculated the relative DNA methylation degree (R_DMeD) of cancer cell *i* in genomic tile *j* (*R_DMeD*_*i,j*_) by dividing the DNA methylation levels of cancer cell *i* in tile *j* by the median DNA methylation levels of the normal cells in tile *j*. The DNA demethylation degree (DDemeD) of cancer cell *i* in tile *j* (*DDemeD*_*i,j*_) was defined as:

$$DDemeD_{i,j} = 1 - R\_DMeD_{i,j}$$

We calculated the Spearman correlation coefficients between the DNA demethylation degree and the distribution densities of histone modifications and LINE-1 and in individual cancer cells across a range of resolutions (0.25–10 Mb).

### NDR calling and TF motif enrichment analysis

First, we searched for single-cell NDRs (scNDRs) in individual cells following the methods of a previous study^18^. In brief, we used a 100-bp sliding window with a step length of 20 bp. The *χ*^2^ test was performed to find the enrichment of significantly higher chromatin accessibility than the genome-wide background. Only windows with *P*-value < 1.0E−15, length > 140 bp and covered GCH sites ≥5 were considered NDRs. Then, we used BEDtools (version 2.17.0)^49^ with the parameter “-d -100” to merge the scNDRs within normal epithelial cells and cancer cells separately. By doing so, scNDRs with >100 bp of overlap in different cells would be merged as a merged NDR (mNDR). Then, we filtered out the mNDRs covered by <11 cells in each group for heterogeneity of each patient or 5% cells in each cell group (11/218 cells in Norm_epi cell group and 53/1077 cells in the cancer cell group) for homogeneity in all patients. Since the GCH site coverage is 14.8%, the standard of “5% cells” mentioned above actually meant “33% cells” that each GCH site may be detected in 53 cells out of 159 cancer cells (1077 cells * 14.8% = 159 cells). The remaining mNDRs in the two cell groups were compared, and the group-specific mNDRs were defined as those that only occurred in one group and did not overlap with the mNDRs of the other group. Next, we used Homer (version 4.11)^50^ to search for TF motif enrichment in the group-specific mNDRs with default parameters. Only known motifs with *P*-value < 1E−10 were considered significantly enriched.

### Gene ontology enrichment analysis

We uploaded single or multiple gene lists onto the website (http://www.metascape.org/) for online analysis. For multiple gene list analysis, gene sets belonging to different groups would be directly compared on both gene contents and enriched items. Clustering of different groups (Fig. 2d) of genes were automatically generated according to similarities of their enriched items.

### Comparison of gene expression with the published data

We extracted UMI count matrix of Ductal 1 cells and Ductal 2 cells from previously published data^11^. After merging, we obtained a count matrix of 22,927 cells with 17,340 genes which were normalized to a TPM matrix subsequently. We used Seurat package in R to integrate these two datasets. Standard pre-processing was performed with 2000 highly variable genes and top 30 PCs. The integrated results were visualized by UMAP plot.

### Comparison of chromatin open sites with the published data

The peak matrix of normal cells (Ductal 1) and cancer cells (Ductal 2) were extracted from previous single-cell ATAC-seq data of PDAC^14^. After filtering peaks present in <5% of cells in corresponding cell types, we obtained 18,664 and 11,466 peaks from normal cells and cancer cells, respectively. Then, we used “bedtools intersect” command to find the overlap region between these peaks and the NDRs of our study. 16,573 and 10,957 peaks from normal cells and cancer cells, respectively, were found to overlap with our NDRs.

### Correlations between DNA methylation and genome copy number

To explore the relationship between copy number variations and methylation levels in subclones, we calculated the DNA methylation levels with the same 2706 variable-length windows as used in the calculation of SCNA. The DNA methylation levels and copy number levels were first averaged across cells in the same subclone. Then we calculated the Pearson correlation coefficients between the DNA methylation level and copy number level across the genomic windows.

### Overall survival analysis

The overall survival analyses of TCGA PAAD (pancreatic adenocarcinoma) were performed using a website server (http://gepia2.cancer-pku.cn/). The high and low expression groups were selected using quartile as the group cutoff.

## Supplementary information

Supplementary Figs. S1-S15

Supplementary Table S1

Supplementary Table S2

Supplementary Table S3

## Data Availability

The raw sequencing data were deposited in The Genome Sequence Archive for Human (GSA-Human) with the accession number: HRA000433.
