# Supplementary material for: Integrated single-cell multiomics analysis reveals novel candidate markers for prognosis in human pancreatic ductal adenocarcinoma
Source: Cell Discov. 2022 Feb 15;8:13. doi: 10.1038/s41421-021-00366-y (PMC8844066; doi:10.1038/s41421-021-00366-y)
Supplement: Supplementary file 1 — Supplementary Figs. S1-S15 [file 41421_2021_366_MOESM1_ESM.pdf]

Figure S1

a

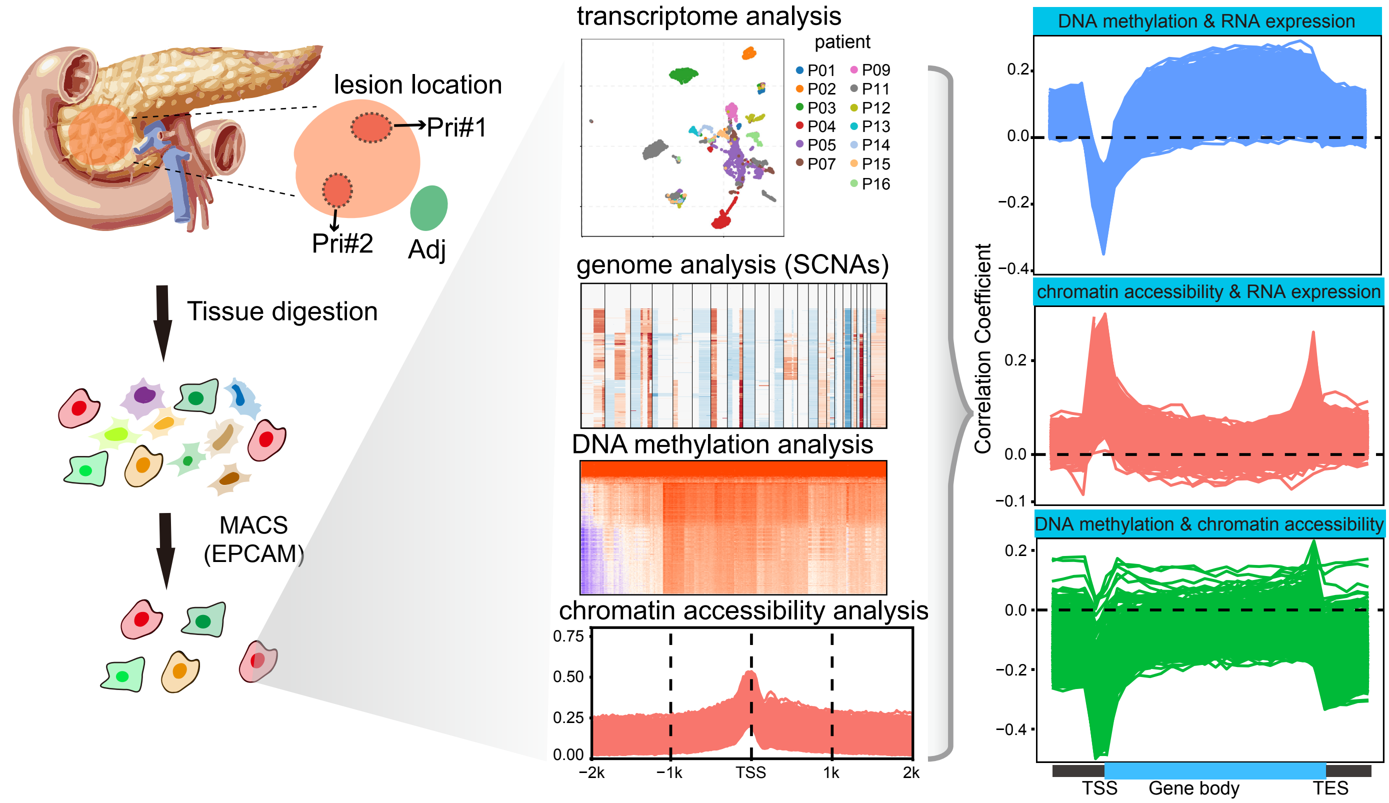

b

Tissue region / Patient ID	Pri #1				Pri #2				Pri #3				Adj		
	No. of cells with RNA	No. of cells with quality passed RNA	No. of cancer cells	No. of Norm_epi cells	No. of cells with RNA	No. of cells with quality passed RNA	No. of cancer cells	No. of Norm_epi cells	No. of cells with RNA	No. of cells with quality passed RNA	No. of cancer cells	No. of Norm_epi cells	No. of cells with RNA	No. of cells with quality passed RNA	No. of Norm_epi cells
P01	96	82	12	12	0	0	0	0	0	0	0	0	0	0	0
P02	16	16	12	0	80	64	37	0	96	93	61	1	0	0	0
P03	192	184	27	0	192	183	23	0	0	0	0	0	0	0	0
P04	192	169	21	16	192	166	33	3	0	0	0	0	0	0	0
P05	480	402	104	45	480	413	176	1	0	0	0	0	0	0	0
P07	48	43	16	16	48	42	17	8	0	0	0	0	96	79	49
P09	96	90	13	0	80	76	10	1	0	0	0	0	32	27	0
P11	192	174	56	21	192	182	132	6	192	182	101	10	96	82	23
P12	48	33	13	0	48	40	23	0	0	0	0	0	48	47	0
P13	48	31	16	0	48	45	26	0	0	0	0	0	0	0	0
P14	48	33	19	0	48	45	29	0	0	0	0	0	0	0	0
P15	48	35	18	2	48	32	25	0	0	0	0	0	24	18	1
P16	48	35	22	3	48	46	35	0	0	0	0	0	48	36	0
Total	1,552	1,327	349	115	1,504	1,334	566	19	288	275	162	11	344	289	73

*A total of 3,225 single cells passed the RNA quality control, among which 1,295 single cells were also passed the DNA methylome and SCNA quality control.

c

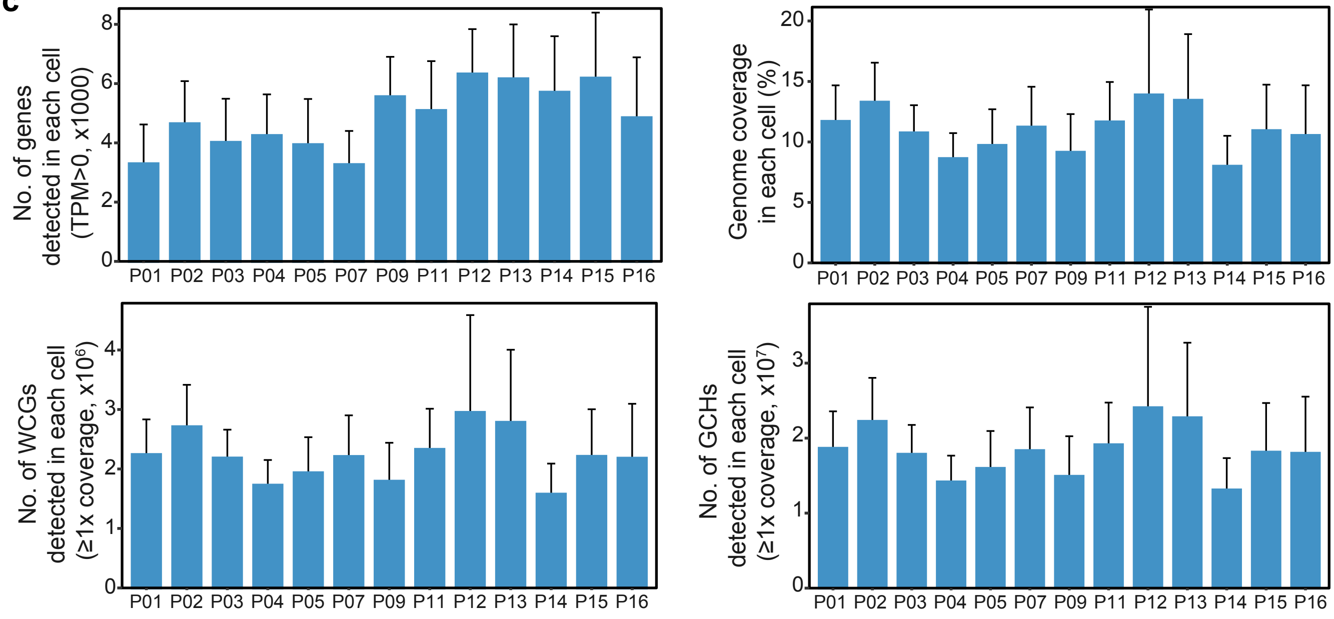

Supplementary Fig. S1 Overview of sampling and data analyses. a Schematics of experimental and bioinformatic approaches to obtain epithelial cells and further perform integrated multiomics analysis. **b** Details of cell numbers from each region in each patient with profiling data of different omics layers. **c** Histogram showing the number of detected genes, WCG sites and GCH sites in each individual cell. The DNA methylome data in each individual cell cover ~10% of the whole genome.

Figure S2

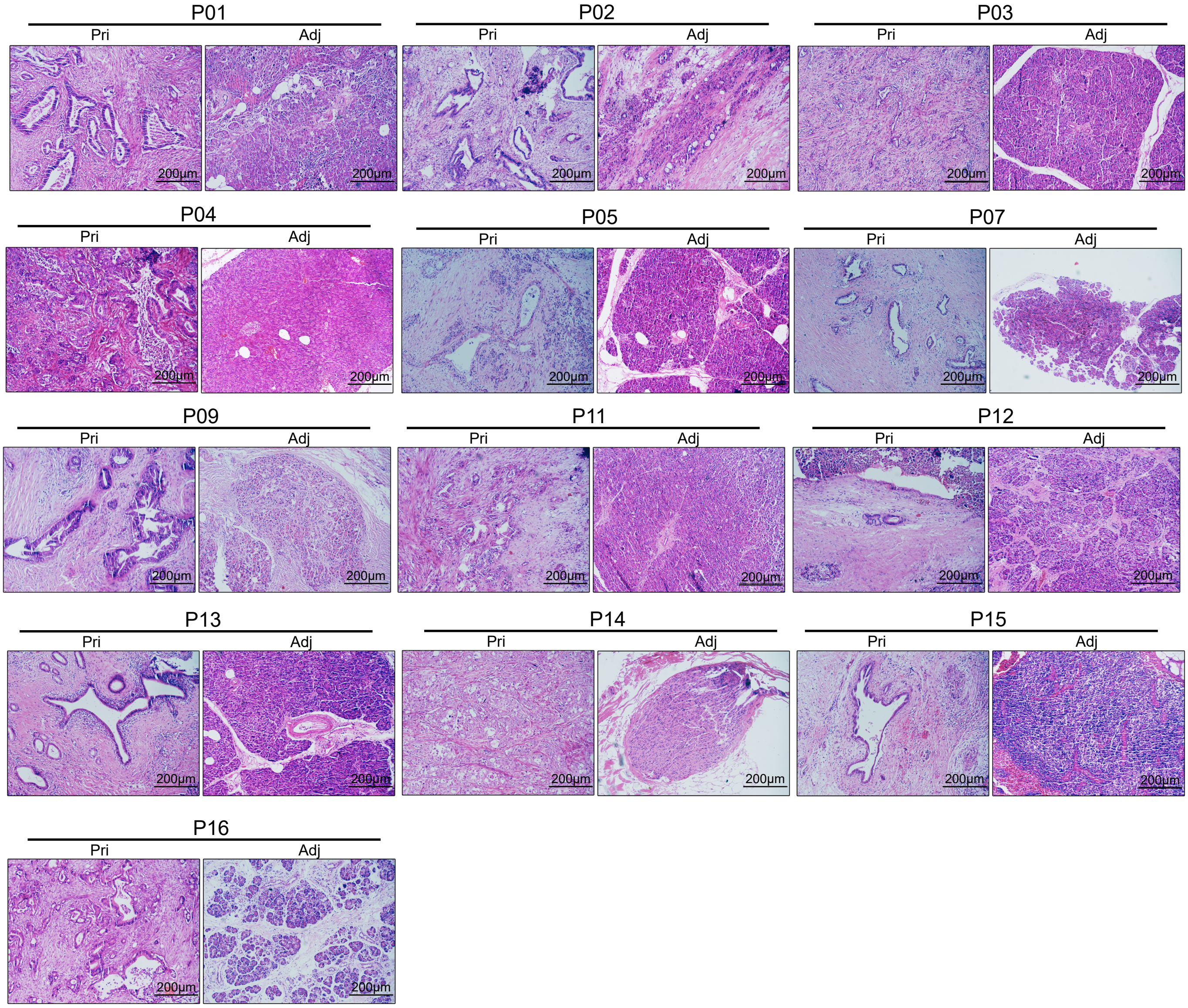
P01
Pri
Adj
P02
Pri
Adj
P03
Pri
Adj
P04
Pri
Adj
P05
Pri
Adj
P07
Pri
Adj
P09
Pri
Adj
P11
Pri
Adj
P12
Pri
Adj
P13
Pri
Adj
P14
Pri
Adj
P15
Pri
Adj
P16
Pri
Adj
200μm

Supplementary Fig. S2 PDAC tumor tissue configuration. Hematoxylin and eosin (H&E) staining of primary tissue and adjacent tissue for each patient we analyzed in this study. The adjacent tissue of P09 shows an abnormal structure with abundant stromal cells, similar to that in tumor tissues.

Figure S3

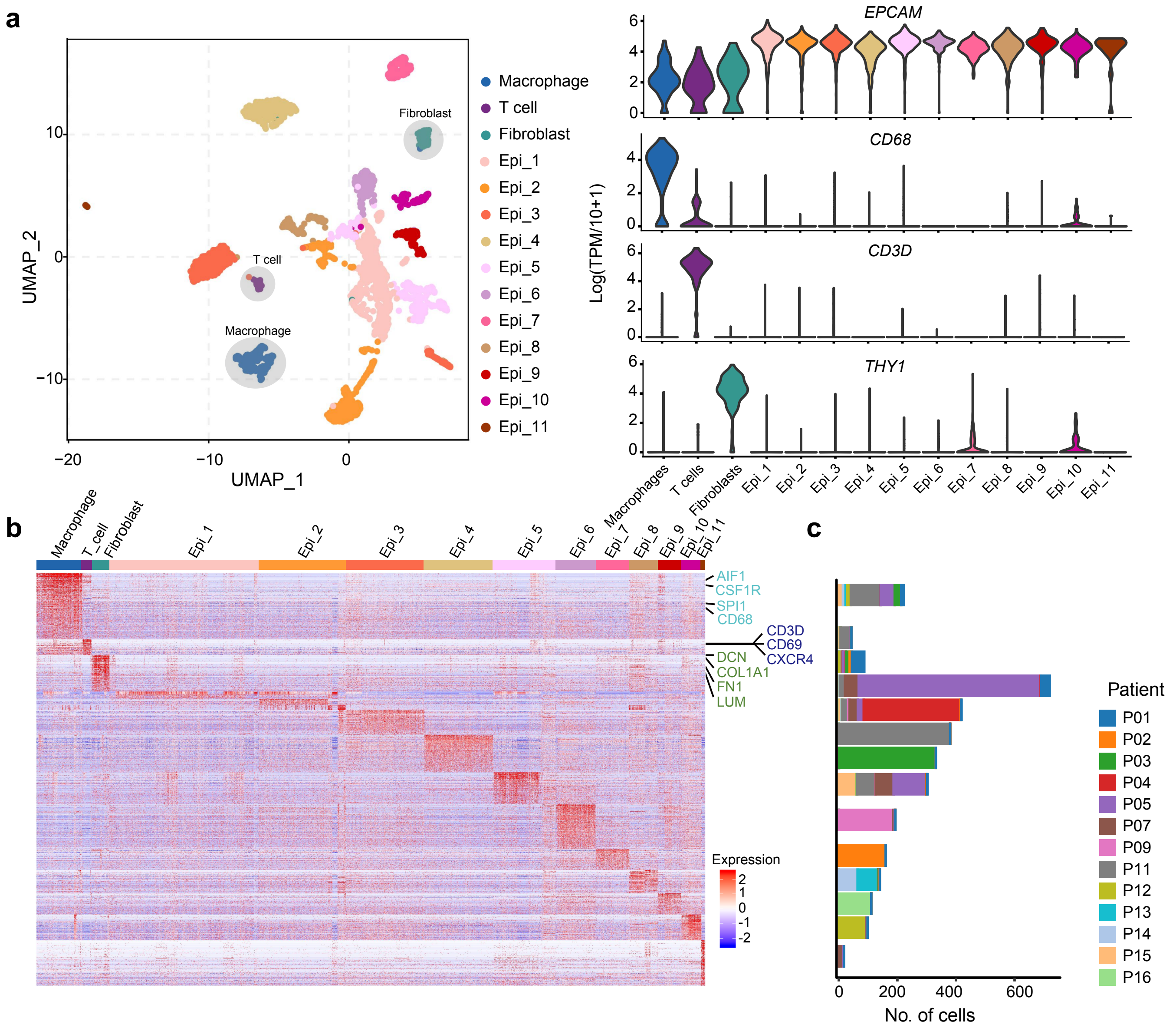
a
Macrophage
T cell
Fibroblast
Epi_1
Epi_2
Epi_3
Epi_4
Epi_5
Epi_6
Epi_7
Epi_8
Epi_9
Epi_10
Epi_11
UMAP_1
UMAP_2
EPCAM
CD68
CD3D
THY1
Log(TPM/10+1)
Macrophages
T cells
Fibroblasts
b
AIF1
CSF1R
SPI1
CD68
CD3D
CD69
CXCR4
DCN
COL1A1
FN1
LUM
Expression
c
Patient
P01
P02
P03
P04
P05
P07
P09
P11
P12
P13
P14
P15
P16
No. of cells

Supplementary Fig. S3 Cell type identification by the single-cell transcriptome aspects of the single-cell multiomics data. a Unsupervised clustering of all cells from the 13 patients using gene expression profiles. The expression levels of the representative cell type marker genes (*EPCAM*-high: epithelial cell; *CD68*-high: macrophage; *CD3D*-high: T cell; *THY1*-high: fibroblast) in each cluster are shown in the violin plot on the right. **b** Heatmap of cluster-specific gene expression further confirming the identification of each cell type. **c** Statistics of cell originations in each cluster corresponding to **b.**

Figure S4

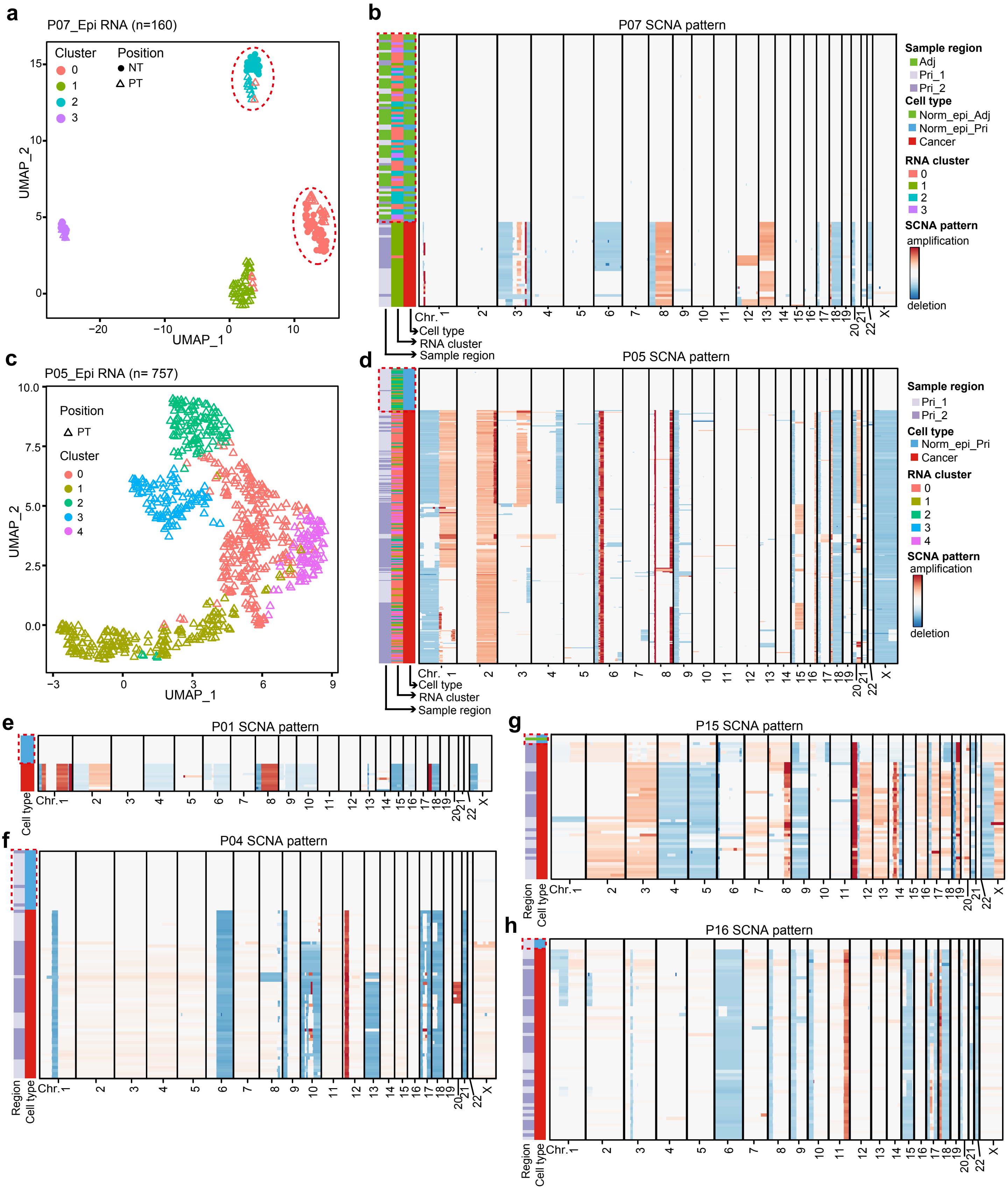

a
P07_Epi RNA (n=160)
Cluster
Position
NT
PT
UMAP_1
UMAP_2
b
P07 SCNA pattern
Sample region
Adj
Pri_1
Pri_2
Cell type
Norm_epi_Adj
Norm_epi_Pri
Cancer
RNA cluster
SCNA pattern
amplification
deletion
Chr.
Cell type
RNA cluster
Sample region
c
P05_Epi RNA (n= 757)
d
P05 SCNA pattern
e
P01 SCNA pattern
f
P04 SCNA pattern
g
P15 SCNA pattern
h
P16 SCNA pattern
Region

Supplementary Fig. S4 Epithelial cells with euploid genomes identified in the primary tumor tissue of each patient. a, **c** Unsupervised clustering of epithelial cells in P07 and P05 using gene expression profiles. The red dashed line indicates that epithelial cells in the primary tumor show similar expression patterns by clustering with those in adjacent tissue. **b**, **d–h** SCNA maps showing a high frequency of subchromosome-scale SCNAs in each PDAC patient. The dashed line and the red arrow indicate epithelial cells in the primary tissue regions.

Figure S5

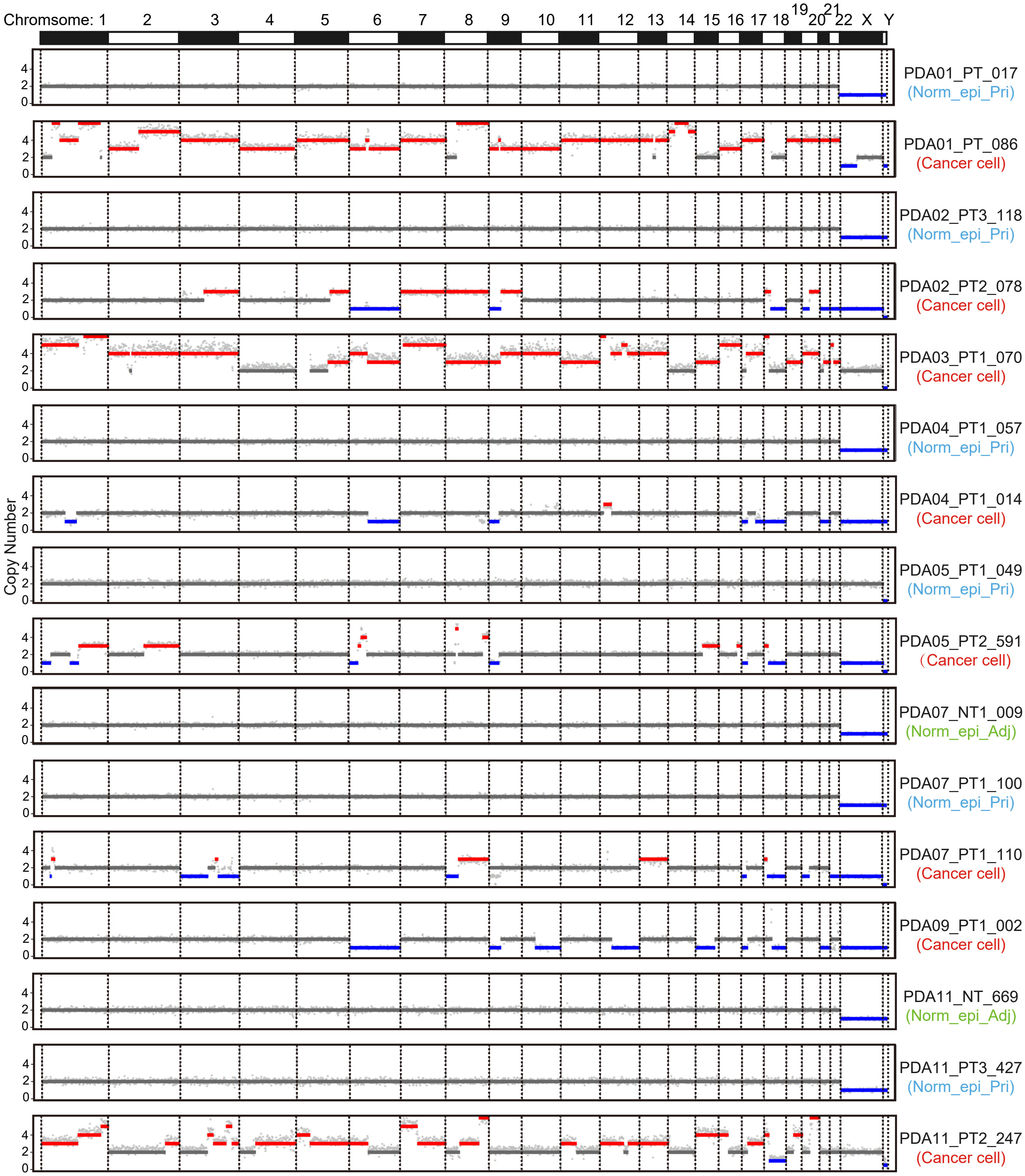
Chromsome: 1
2
3
4
5
6
7
8
9
10
11
12
13
14
15
16
17
18
19
20
21
22
X
Y
Copy Number
0
2
4
PDA01_PT_017
(Norm_epi_Pri)
PDA01_PT_086
(Cancer cell)
PDA02_PT3_118
(Norm_epi_Pri)
PDA02_PT2_078
(Cancer cell)
PDA03_PT1_070
(Cancer cell)
PDA04_PT1_057
(Norm_epi_Pri)
PDA04_PT1_014
(Cancer cell)
PDA05_PT1_049
(Norm_epi_Pri)
PDA05_PT2_591
（Cancer cell)
PDA07_NT1_009
(Norm_epi_Adj)
PDA07_PT1_100
(Norm_epi_Pri)
PDA07_PT1_110
(Cancer cell)
PDA09_PT1_002
(Cancer cell)
PDA11_NT_669
(Norm_epi_Adj)
PDA11_PT3_427
(Norm_epi_Pri)
PDA11_PT2_247
(Cancer cell)

Supplementary Fig. S5 Representative SCNA results for each cell type in the patients. Single Norm_epi cells have euploid genomes, and cancer cells show diversified subchromosome-scale SCNAs across different chromosomes in different patients.

Figure S6

a

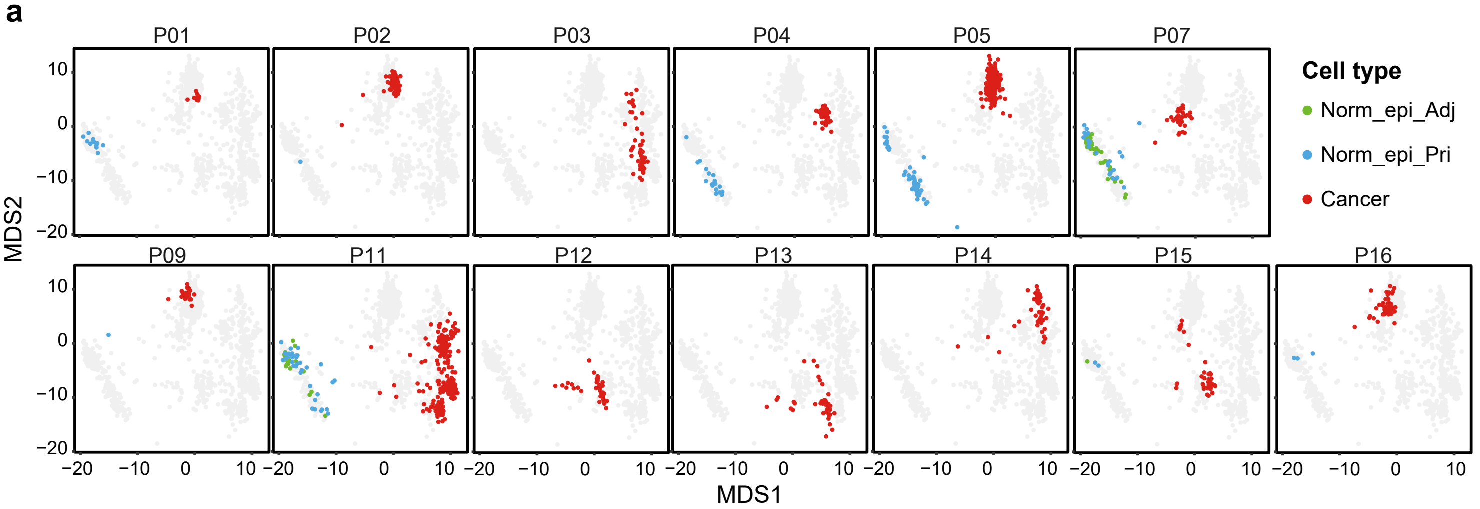
P01
P02
P03
P04
P05
P07
P09
P11
P12
P13
P14
P15
P16
Cell type
Norm_epi_Adj
Norm_epi_Pri
Cancer
MDS2
MDS1
10
0
−10
−20
−20
−10
0
10

b

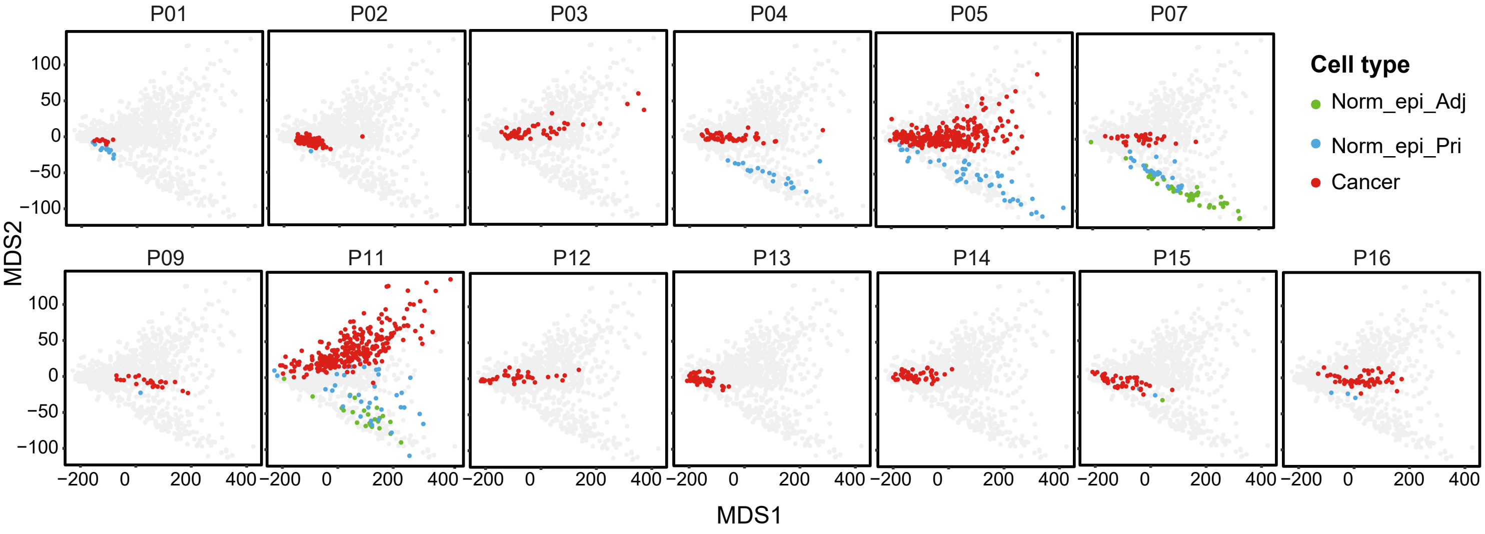
P01
P02
P03
P04
P05
P07
P09
P11
P12
P13
P14
P15
P16
Cell type
Norm_epi_Adj
Norm_epi_Pri
Cancer
MDS2
MDS1
100
50
0
−50
−100
−200
0
200
400

Supplementary Fig. S6 Norm_epi_Pri cells can be separated from cancer cells according to the GCH and WCG methylation levels in promoter region. a, **b** Multidimensional scaling analysis of promoter DNA methylation levels **(a)** and normalized chromatin accessibility levels **(b)** for all epithelial cells in each patient.

Figure S7

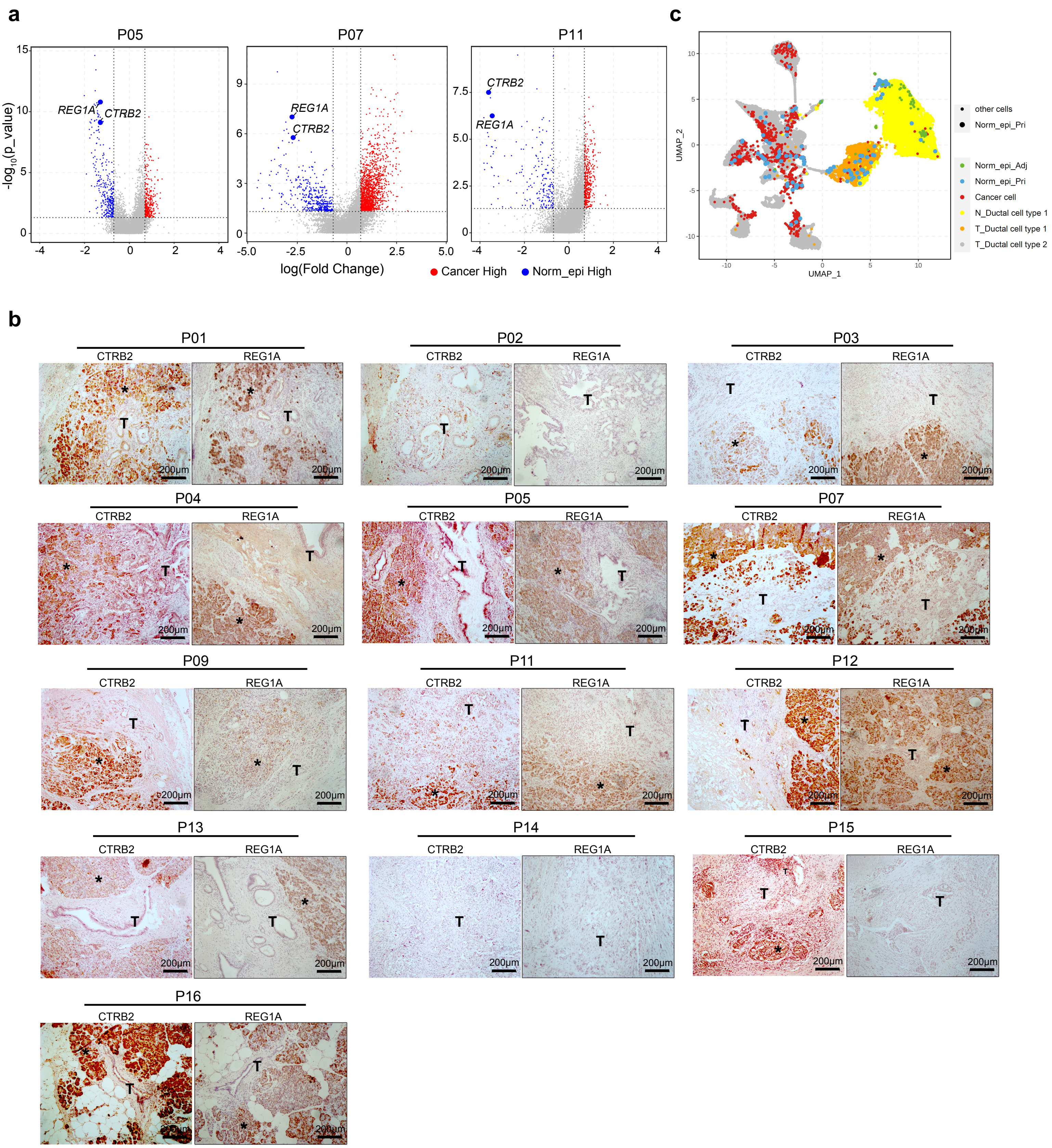
a
P05
P07
P11
-log10(p_value)
log(Fold Change)
REG1A
CTRB2
Cancer High
Norm_epi High
c
UMAP_1
UMAP_2
other cells
Norm_epi_Pri
Norm_epi_Adj
Norm_epi_Pri
Cancer cell
N_Ductal cell type 1
T_Ductal cell type 1
T_Ductal cell type 2
b
P01
P02
P03
P04
P05
P07
P09
P11
P12
P13
P14
P15
P16
CTRB2
REG1A
200μm

Supplementary Fig. S7 Characteristics of Norm_epi_Pri cells. a The volcano plots of DEGs (*P*-value < 0.05, Fold Change > 2 or < 0.5) between cancer cells and Norm_epi cells in P05, P07 and P11. *REG1A* and *CTRB2* were highly detected in the Norm_epi cells of each patient. **b** The UMAP plot of epithelium data integration between this study and Peng *et al.* (2019). **c** Immunostaining of *REG1A* and *CTRB2* in the tumor tissue of each patient. Only P02 and P14 were not detected with positive signals.

Figure S8

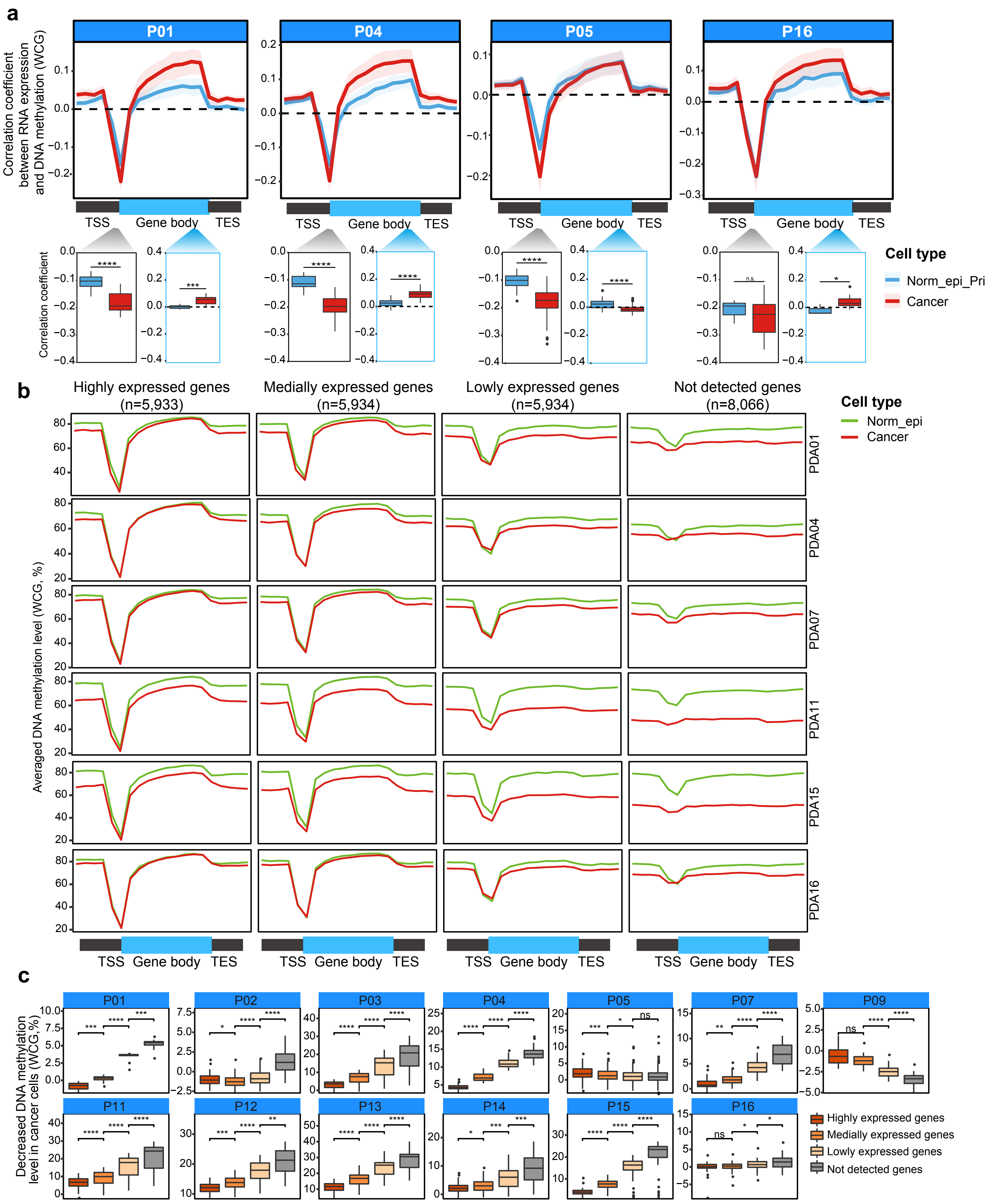
a
P01
P04
P05
P16
Correlation coefficient between RNA expression and DNA methylation (WCG)
TSS
Gene body
TES
Correlation coefficient
Cell type
Norm_epi_Pri
Cancer
b
Highly expressed genes (n=5,933)
Medially expressed genes (n=5,934)
Lowly expressed genes (n=5,934)
Not detected genes (n=8,066)
Cell type
Norm_epi
Cancer
PDA01
PDA04
PDA07
PDA11
PDA15
PDA16
Averaged DNA methylation level (WCG, %)
c
P01
P02
P03
P04
P05
P07
P09
P11
P12
P13
P14
P15
P16
Decreased DNA methylation level in cancer cells (WCG,%)
Highly expressed genes
Medially expressed genes
Lowly expressed genes
Not detected genes

Supplementary Fig. S8 DNA methylation shows a stronger correlation with RNA expression in cancer cells. a Spearman correlations between DNA methylation levels across gene bodies (including 15 kb flanking regions) and corresponding RNA expression levels in each cell type in representative patients. The boxplots at the bottom show the statistical test results between cancer cells and Norm_epi cells using the Wilcoxon rank sum test. The promoter regions are from -1 kb to +0.5 kb around the TSS, and the gene body regions are +2 kb from the TSS to the TES. n.s., no significance; *$P < 0.05$; **$P < 0.01$; ***$P < 0.001$; ****$P < 0.0001$. **b** Averaged DNA methylation levels across gene bodies in different groups of genes. The genes are grouped according to their expression levels in Norm_epi cells. **c** Boxplots showing the decreased DNA methylation levels in cancer cells compared to Norm_epi cells in each group of genes. All 13 patients showed significantly stronger DNA demethylation in lowly and not expressed genes. P09 showed a reversed pattern.

Figure S9

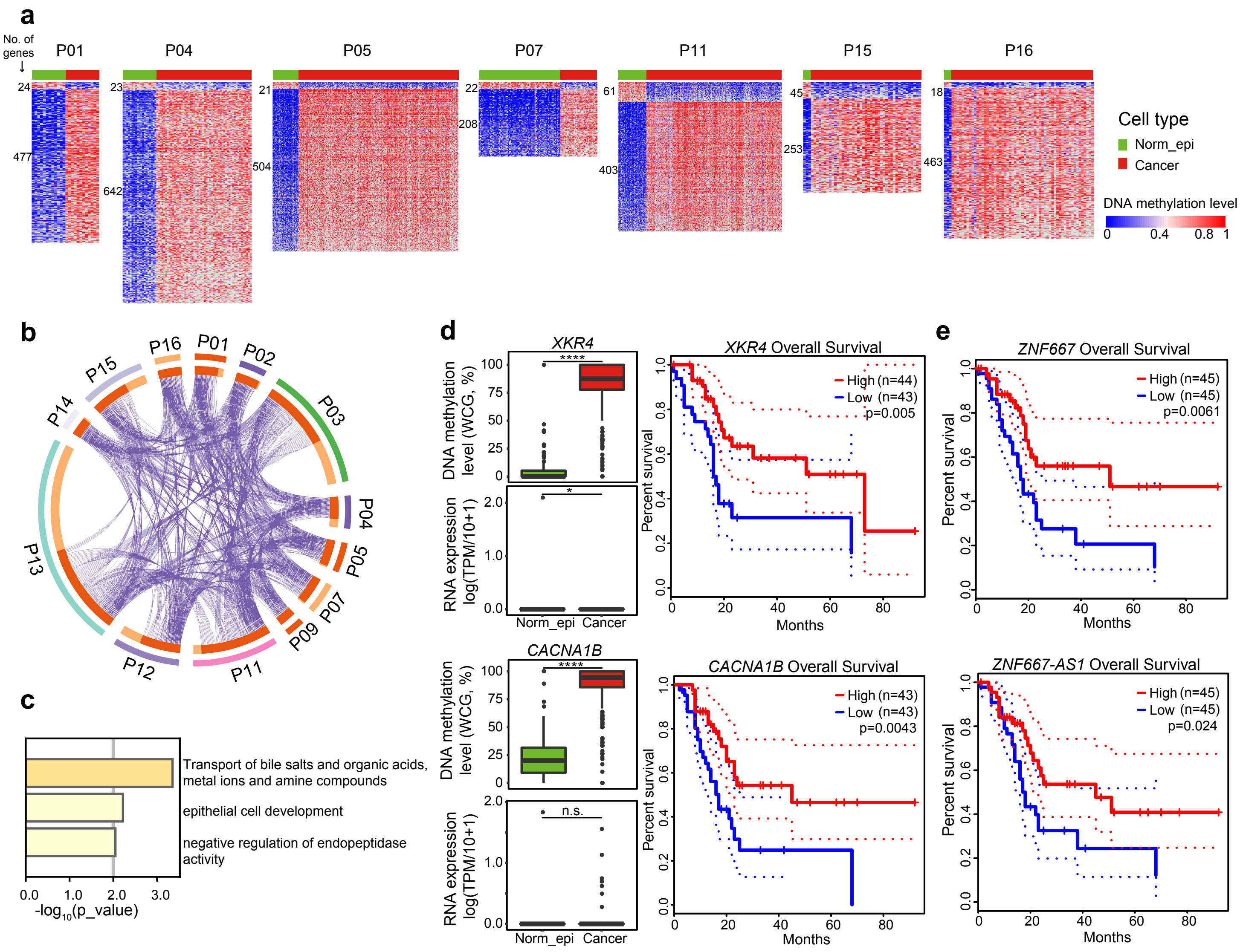

a
No. of genes
P01
P04
P05
P07
P11
P15
P16
24
477
23
642
21
504
22
208
61
403
45
253
18
463
Cell type
Norm_epi
Cancer
DNA methylation level
0
0.4
0.8
1
b
P16
P01
P02
P03
P04
P05
P07
P09
P11
P12
P13
P14
P15
c
Transport of bile salts and organic acids, metal ions and amine compounds
epithelial cell development
negative regulation of endopeptidase activity
0.0
1.0
2.0
3.0
-log10(p_value)
d
XKR4
DNA methylation level (WCG, %)
RNA expression log(TPM/10+1)
****
*
Norm_epi
Cancer
XKR4 Overall Survival
High (n=44)
Low (n=43)
p=0.005
Percent survival
Months
CACNA1B
****
n.s.
CACNA1B Overall Survival
High (n=43)
Low (n=43)
p=0.0043
e
ZNF667 Overall Survival
High (n=45)
Low (n=45)
p=0.0061
ZNF667-AS1 Overall Survival
High (n=45)
Low (n=45)
p=0.024

Supplementary Fig. S9 Promoter regions in cancer cells show global DNA hypermethylation. **a** Heatmaps showing the DNA methylation levels of DMPs (differentially methylated promoters) between cancer cells in each patient and all Norm_epi cells. Patients with over 3 Norm_epi cells are shown. **b** Overlap of hypomethylated promoters across all patients. The purple lines indicate shared genes between different patients. **c** Enriched biological processes for hypomethylated genes in at least 3 patients. **d** The representative genes, *XKR4* and *CACNA1B*, showing increased promoter DNA methylation levels, were significantly correlated with overall survival of PDAC patients recorded in the TCGA database. **e** The two novel candidate genes *ZNF667* and *ZNF667-AS1* are significantly correlated with overall survival of the PDAC patients in the TCGA database.

Figure S10

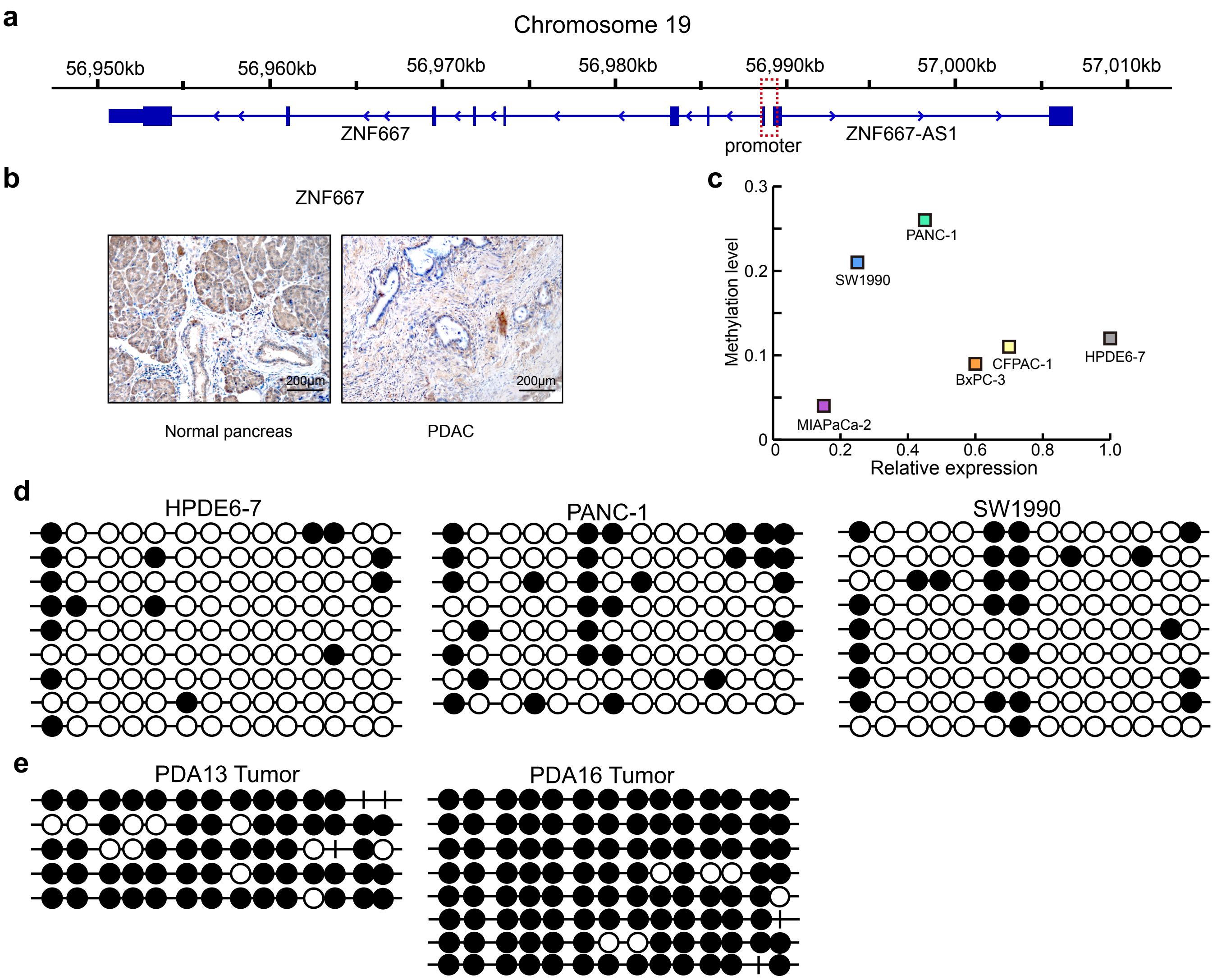
a
Chromosome 19
56,950kb
56,960kb
56,970kb
56,980kb
56,990kb
57,000kb
57,010kb
ZNF667
promoter
ZNF667-AS1
b
ZNF667
200μm
200μm
Normal pancreas
PDAC
c
Methylation level
Relative expression
PANC-1
SW1990
CFPAC-1
HPDE6-7
BxPC-3
MIAPaCa-2
d
HPDE6-7
PANC-1
SW1990
e
PDA13 Tumor
PDA16 Tumor

Supplementary Fig. S10 Abundance of *ZNF667* and *ZNF667-AS1* in pancreas cancer cell lines. **a** The genome locations of *ZNF667* and *ZNF667-AS1.* **b** Immunohistochemistry of ZNF667 showing variations of abundance in normal pancreas and PDAC using the tissue microarray analysis. Scale bar, 200 μm. **c** The methylation level and relative expression of ZNF667-AS1 in each cell line. All *in vitro* cell types showed lower methylation level than that we detected in *in vivo* PDAC cancer cells. **d, e** Plots showing the methylation pattern of a CpG island containing 14 CpG sites in the *ZNF667-AS1* promoter region in the normal cell line HPDE6-7, the two pancreas cancer cell lines PANC-1 and SW1990 (**d**), and two tumor samples (**e**).

Figure S11

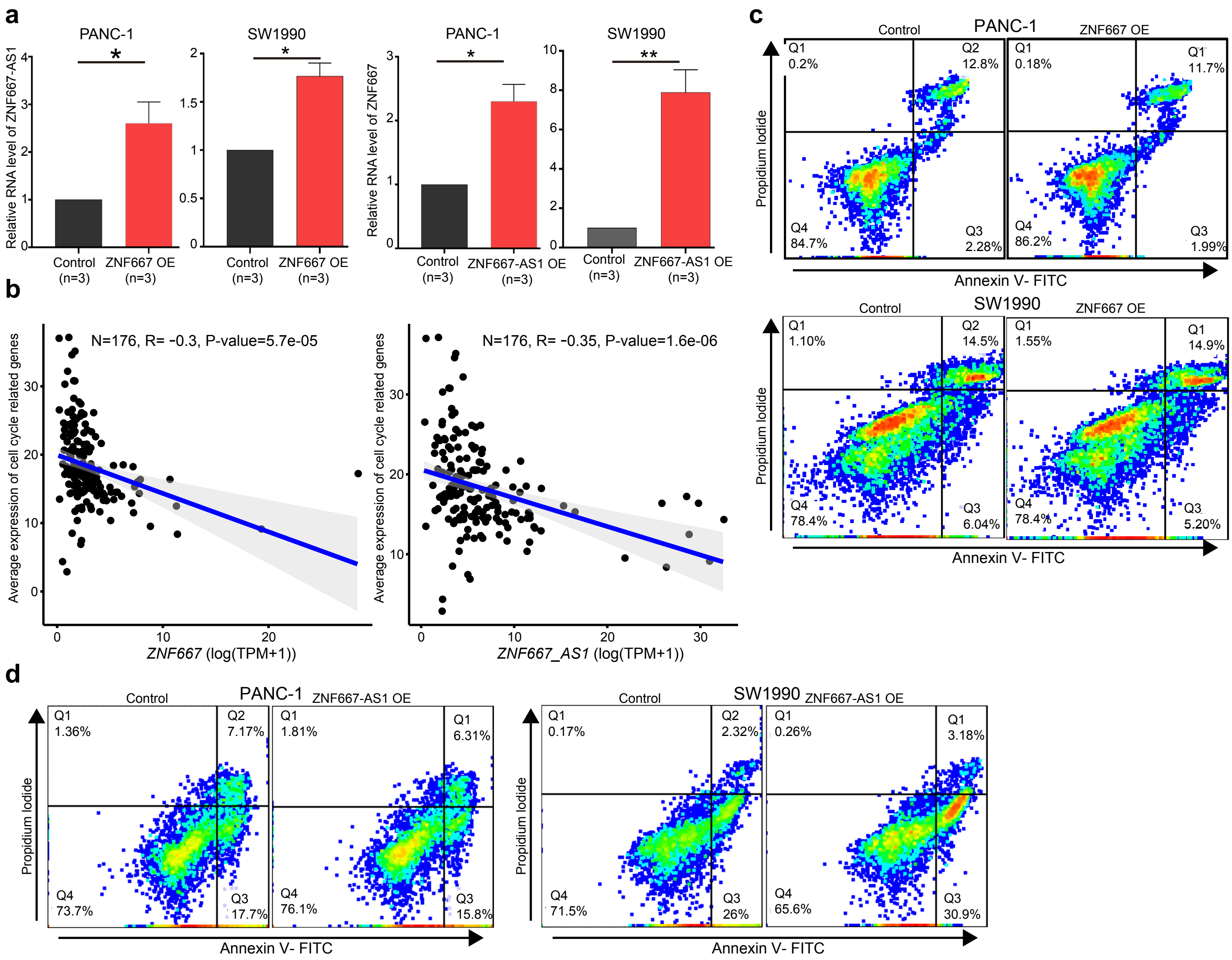

a
PANC-1
SW1990
PANC-1
SW1990
Relative RNA level of ZNF667-AS1
Relative RNA level of ZNF667
Control (n=3)
ZNF667 OE (n=3)
ZNF667-AS1 OE (n=3)
b
N=176, R= -0.3, P-value=5.7e-05
N=176, R= -0.35, P-value=1.6e-06
Average expression of cell cycle related genes
ZNF667 (log(TPM+1))
ZNF667_AS1 (log(TPM+1))
c
PANC-1
Control
ZNF667 OE
Q1 0.2%
Q2 12.8%
Q3 2.28%
Q4 84.7%
Q1 0.18%
Q1 11.7%
Q3 1.99%
Q4 86.2%
Propidium Iodide
Annexin V- FITC
SW1990
Control
ZNF667 OE
Q1 1.10%
Q2 14.5%
Q3 6.04%
Q4 78.4%
Q1 1.55%
Q1 14.9%
Q3 5.20%
Q4 78.4%
Propidium Iodide
Annexin V- FITC
d
PANC-1
Control
ZNF667-AS1 OE
Q1 1.36%
Q2 7.17%
Q3 17.7%
Q4 73.7%
Q1 1.81%
Q1 6.31%
Q3 15.8%
Q4 76.1%
SW1990
Control
ZNF667-AS1 OE
Q1 0.17%
Q2 2.32%
Q3 26%
Q4 71.5%
Q1 0.26%
Q1 3.18%
Q3 30.9%
Q4 65.6%
Propidium Iodide
Annexin V- FITC

Supplementary Fig. S11 Functional validations of *ZNF667* and *ZNF667-AS1* in the pancreas cancer cell lines. a Boxplot showing the relative changes in the expression levels of ZNF667 when overexpressing ZNF667-AS1, and the other way around, in the two cancer cell lines. **b** Correlation of the expression levels between *ZNF667, ZNF667-AS1* and the cell cycle genes according to the transcriptome data in TCGA PAAD patients. **c, d** FACS record of apoptosis detection in the two pancreas cell lines after overexpression *ZNF667* and *ZNF667-AS1*.

Figure S12

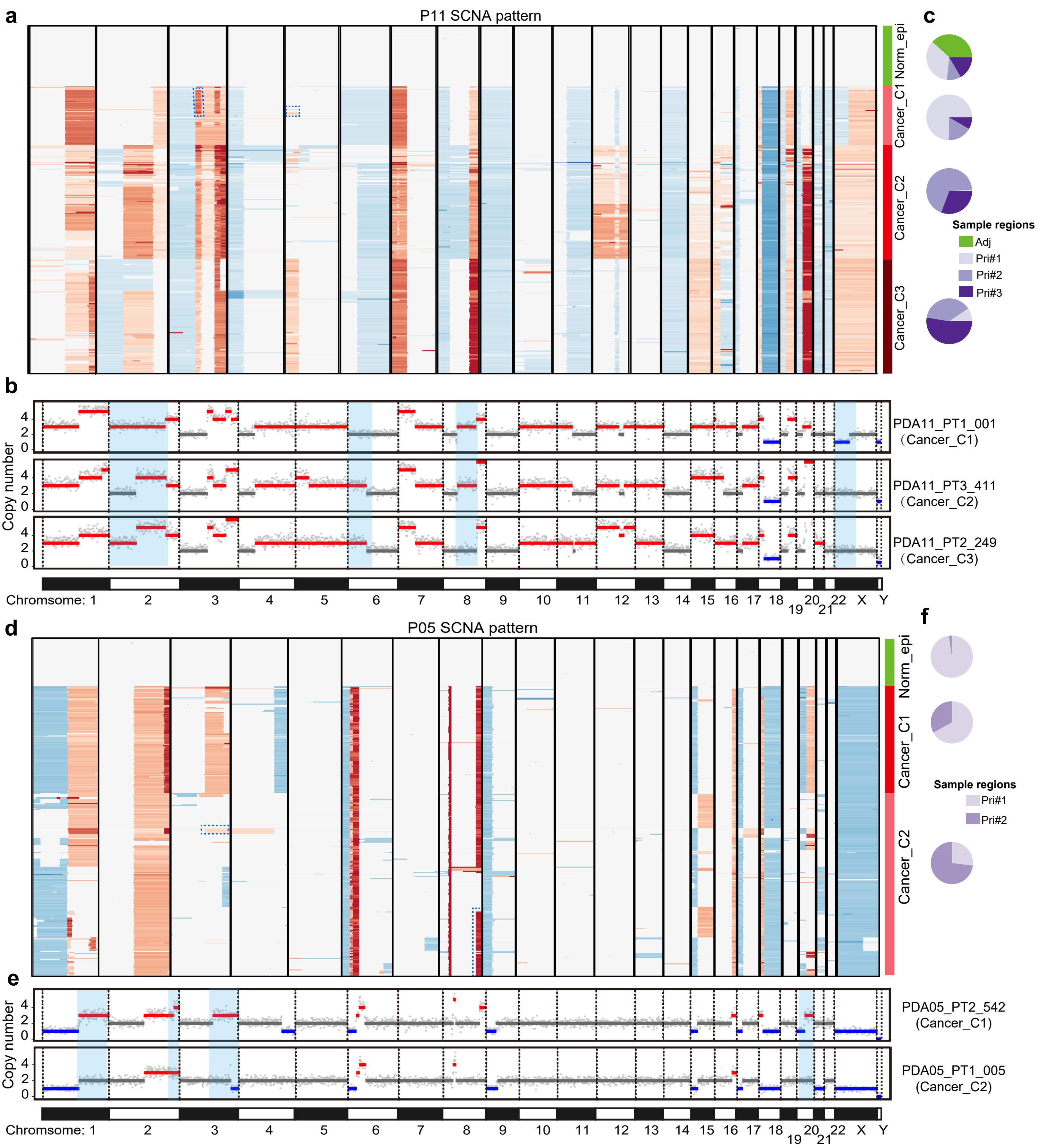
a
P11 SCNA pattern
Norm_epi
Cancer_C1
Cancer_C2
Cancer_C3
c
Sample regions
Adj
Pri#1
Pri#2
Pri#3
b
Copy number
PDA11_PT1_001
(Cancer_C1)
PDA11_PT3_411
(Cancer_C2)
PDA11_PT2_249
(Cancer_C3)
Chromsome: 1 2 3 4 5 6 7 8 9 10 11 12 13 14 15 16 17 18 19 20 21 22 X Y
d
P05 SCNA pattern
Norm_epi
Cancer_C1
Cancer_C2
f
Sample regions
Pri#1
Pri#2
e
Copy number
PDA05_PT2_542
(Cancer_C1)
PDA05_PT1_005
(Cancer_C2)
Chromsome: 1 2 3 4 5 6 7 8 9 10 11 12 13 14 15 16 17 18 19 20 21 22 X Y

Supplementary Fig. S12 Global SCNA patterns indicate subclones in P05 and P11. **a, d** The global SCNA patterns of P05 and P11 (each with > 200 cancer cells) and subclones indicated on the right. **b, e** Examples of single-cell SCNA patterns in each subclone. **c, f** Pie charts showing the regional compositions in each subclone of cells.

Figure S13

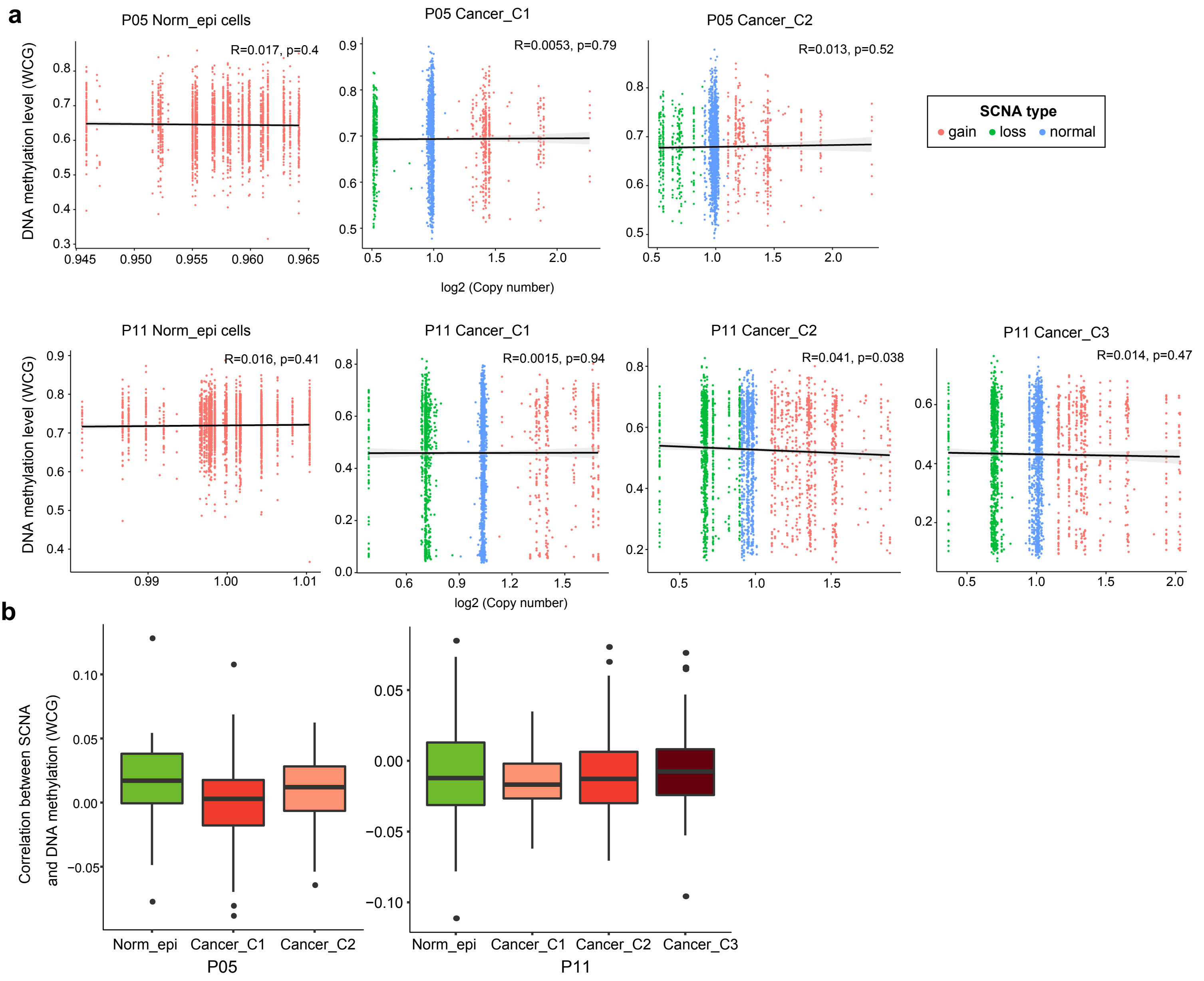
a
P05 Norm_epi cells
R=0.017, p=0.4
P05 Cancer_C1
R=0.0053, p=0.79
P05 Cancer_C2
R=0.013, p=0.52
SCNA type
gain loss normal
DNA methylation level (WCG)
log2 (Copy number)
P11 Norm_epi cells
R=0.016, p=0.41
P11 Cancer_C1
R=0.0015, p=0.94
P11 Cancer_C2
R=0.041, p=0.038
P11 Cancer_C3
R=0.014, p=0.47
b
Correlation between SCNA and DNA methylation (WCG)
Norm_epi
Cancer_C1
Cancer_C2
Cancer_C3
P05
P11

Supplementary Fig. S13 Correlations between SCNA and DNA methylation level. **a** The DNA methylation level in each 1 Mb bin belonging to different SCNA types in every subclones of cells in P05 and P11 patients. **b** Boxplot showing the distribution of correlation values between the genome copy number and the DNA methylation level in each cell subclones.

Figure S14

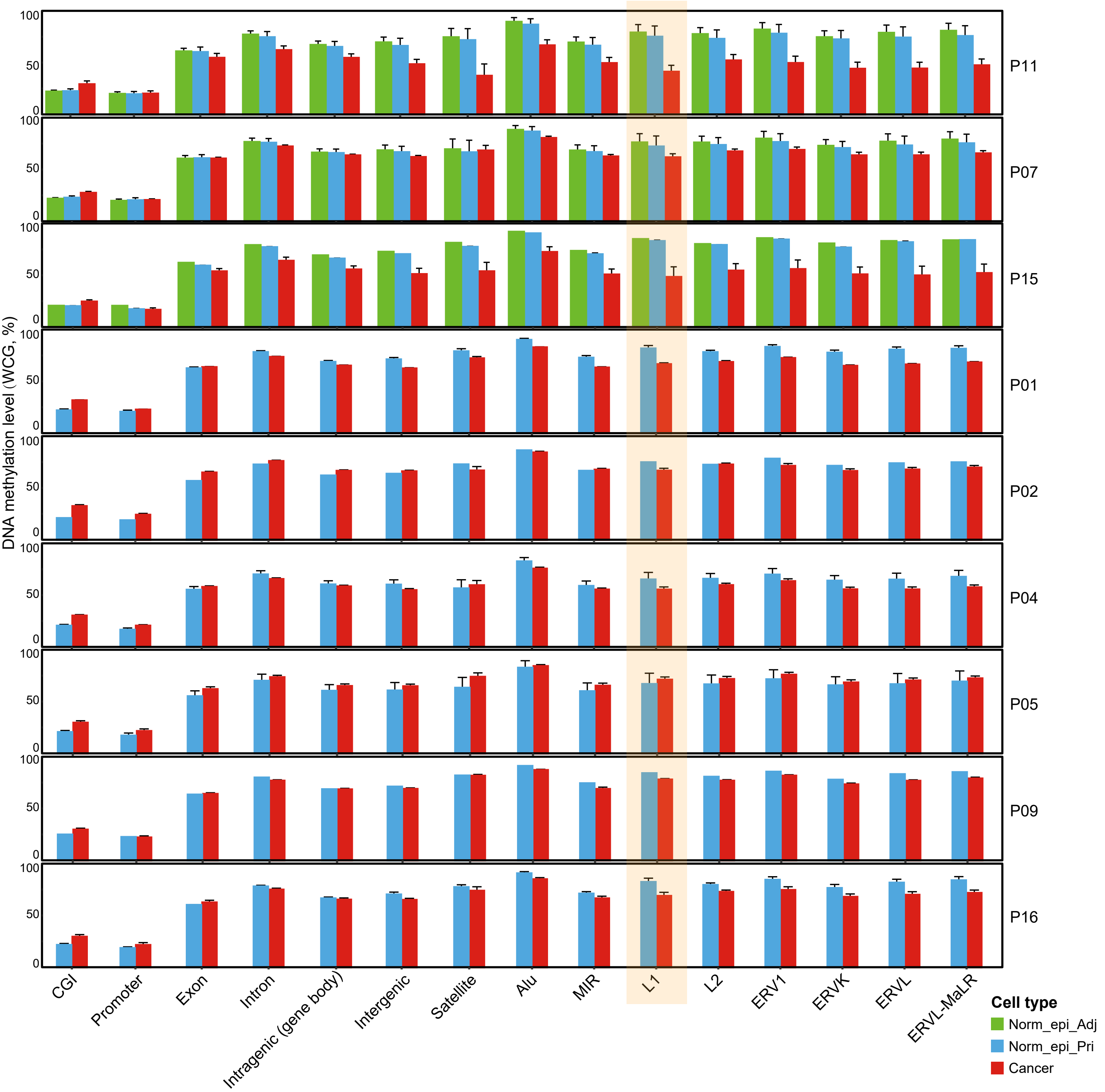

DNA methylation level (WCG, %)
100
50
0
P11
P07
P15
P01
P02
P04
P05
P09
P16
CGI
Promoter
Exon
Intron
Intragenic (gene body)
Intergenic
Satellite
Alu
MIR
L1
L2
ERV1
ERVK
ERVL
ERVL-MaLR
Cell type
Norm_epi_Adj
Norm_epi_Pri
Cancer

Supplementary Fig. S14 Genome-wide DNA demethylation in cancer cells is strongly enriched in heterochromatin regions. Histogram showing the absolute DNA methylation levels of repeat elements and other genomic elements of different cell types in each patient (each with over 3 Norm_epi cells).

Figure S15

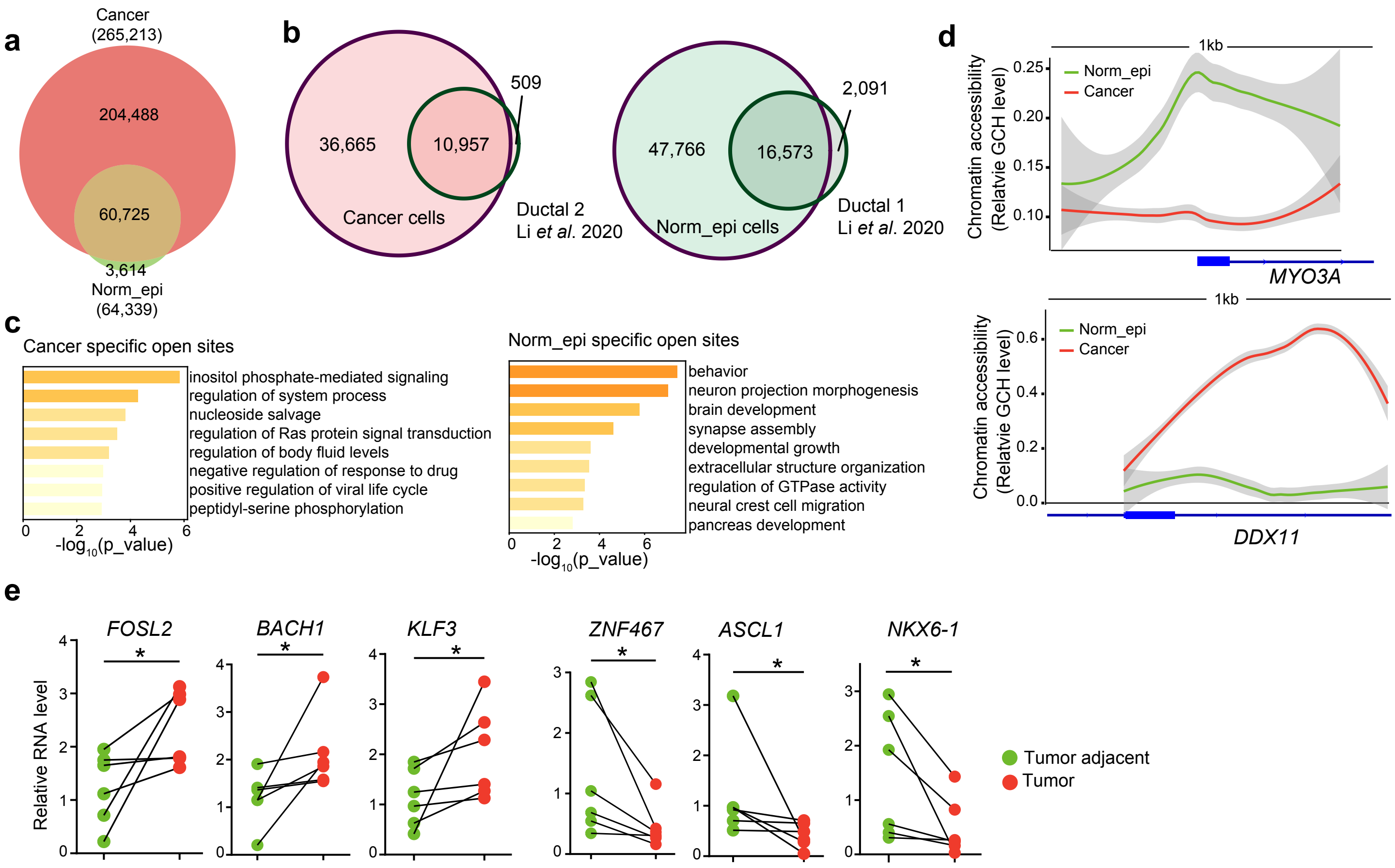

a
Cancer
(265,213)
204,488
60,725
3,614
Norm_epi
(64,339)
b
36,665
10,957
509
Cancer cells
Ductal 2
Li et al. 2020
47,766
16,573
2,091
Norm_epi cells
Ductal 1
Li et al. 2020
c
Cancer specific open sites
inositol phosphate-mediated signaling
regulation of system process
nucleoside salvage
regulation of Ras protein signal transduction
regulation of body fluid levels
negative regulation of response to drug
positive regulation of viral life cycle
peptidyl-serine phosphorylation
-log10(p_value)
Norm_epi specific open sites
behavior
neuron projection morphogenesis
brain development
synapse assembly
developmental growth
extracellular structure organization
regulation of GTPase activity
neural crest cell migration
pancreas development
-log10(p_value)
d
1kb
Norm_epi
Cancer
Chromatin accessibility
(Relatvie GCH level)
MYO3A
1kb
Norm_epi
Cancer
Chromatin accessibility
(Relatvie GCH level)
DDX11
e
Relative RNA level
FOSL2
BACH1
KLF3
ZNF467
ASCL1
NKX6-1
*
Tumor adjacent
Tumor

Supplementary Fig. S15 Chromatin accessibility features in PDAC cells. a Venn diagram indicating the merged NDR numbers in cancer cells and Norm_epi cells. Merged NDRs in two types of cells were defined with two standards: i) NDRs were merged when they had at least a 100-bp overlap; ii) Each merged NDR was detected in at least 11 cells in each cell group. **b** Venn diagram showing the overlap of chromatin open sites identified in our dataset and the previous study by Li *et al.* (2021), respectively, in cancer cells and normal cells. **c** The enriched biological processes for the top 200 genes detected with cell type-specific chromatin open status. **d** Representative NDRs for the cancer cells and Norm_epi cells. **e** RT-qPCR results using the pairwise tumor and tumor-adjacent samples (n = 6) showing the candidate TFs higher expressed in the suspected cell types. *P < 0.05, Wilcoxon matched-pairs signed rank test.
